# Socioeconomic and geographic variation in coverage of health insurance across India

**DOI:** 10.3389/fpubh.2023.1160088

**Published:** 2023-07-10

**Authors:** Mayanka Ambade, Sunil Rajpal, Rockli Kim, S. V. Subramanian

**Affiliations:** ^1^Laxmi Mittal and Family South Asia Institute, Harvard University, India Office, New Delhi, India; ^2^Department of Economics, FLAME University, Pune, Maharashtra, India; ^3^Interdisciplinary Program in Precision Public Health, Department of Public Health Sciences, Graduate School of Korea University, Seoul, Republic of Korea; ^4^Division of Health Policy and Management, College of Health Science, Korea University, Seoul, Republic of Korea; ^5^Harvard Center for Population and Development Studies, Cambridge, MA, United States; ^6^Department of Social and Behavioral Sciences, Harvard T. H. Chan School of Public Health, Boston, MA, United States

**Keywords:** health insurance, India, geographic variation, National Family Health Survey, multilevel modelling

## Abstract

**Introduction:**

In India, regular monitoring of health insurance at district levels (the most essential administrative unit) is important for its effective uptake to contain the high out of pocket health expenditures. Given that the last individual data on health insurance coverage at district levels in India was in 2016, we update the evidence using the latest round of the National Family Health Survey conducted in 2019-2021.

**Methods:**

We use the unit records of households from the latest round (2021) of the nationally representative National Family Health Survey to calculate the weighted percentage (and 95% CI) of households with at least one member covered by any form of health insurance and its types across socio-economic characteristics and geographies of India. Further, we used a random intercept logistic regression to measure the variation in coverage across communities, district and state. Such household level study of coverage is helpful as it represents awareness and outreach for at least one member, which can percolate easily to the entire household with further interventions.

**Results:**

We found that only 2/5^th^ of households in India had insurance coverage for at least one of its members, with vast geographic variation emphasizing need for aggressive expansion. About 15.5% were covered by national schemes, 47.1% by state health scheme, 13.2% by employer provided health insurance, 3.3% had purchased health insurance privately and 25.6% were covered by other health insurance schemes (not covered above). About 30.5% of the total variation in coverage was attributable to state, 2.7% to districts and 9.5% to clusters. Household size, gender, marital status and education of household head show weak gradient for coverage under “any” insurance.

**Discussion:**

Despite substantial increase in population eligible for state sponsored health insurance and rise in private health insurance companies, nearly 60% of families do not have a single person covered under any health insurance scheme. Further, the existing coverage is fragmented, with significant rural/urban and geographic variation within districts. It is essential to consider these disparities and adopt rigorous place-based interventions for improving health insurance coverage.

## Introduction

Low- and Middle-Income countries (LMICs), unlike developed nations, have inadequate and fragmented resource pools and incomplete coverage of social insurance (1). Thus, the health systems of these countries are characterised by high out-of-pocket payments such that 48.2% of total health care expenditure for 2018–19 in India was out-of-pocket (1, 2). This translates to 16% of households in India spending more than 10% of their disposable household income on health care alone (3). In the absence of insurance coverage, 17.2% of rural and 12.7% of urban households resorted to distress modes of financing such as borrowing, and asset selling (4).

Often in economies like India’s, universal health coverage is hoped to be achieved through broader health insurance schemes, i.e., social, private, or community insurance, that covers the socio-economically poor (1). Larger coverage of Public Funded Health Insurances (PFHI) may prove a viable solution, given the high and heterogeneous populace in India. Therefore, between 1999 and 2017, India established multiple publicly financed health insurance (PFHI) schemes (5–7). Some of these schemes are very ambitious, such that USD 587 million were allotted for Rashtriya Swashtya Bima Yojana (RSBY) since its launch (8), and Pradhan Mantri Jan Aarogya Yojana (PMJAY) accounted for USD 782 million in 2021-22 (9). Yet, the financial protection to households from health care costs continues to remain substandard (5, 8, 10–12), despite 9% and 5.8% of Total Health Expenditure (of India in 2017-18) being spent on social insurance schemes and private insurances, respectively (2). Systematic review (7) and individual studies (8, 11, 12) in India show no reduction in the prevalence of out of pocket expenditure (OOPE), despite some increase in health insurance coverage. In fact, some studies have observed an increase in OOPE among those covered by RSBY (13, 14).

Studies suggest identifying “leakages” in converting the population targeted for health insurance into beneficiaries, to understand the driving factors behind the under-performance of healthcare insurance schemes (6, 8, 13, 15, 16). These conversion losses are often noted at the stage of enrolment (15, 16). A study of RSBY conducted in 22 districts of Maharashtra (15) found that 78.4% of the eligible population were not enrolled in the scheme. Other RSBY studies across the country have noted an enrolment rate of 28–46% among eligible populations in the scheme’s early years, but with no significant improvement later on (16). Evidence from nationally representative data shows that government-funded health insurance schemes cover only 9.5% of the eligible population among the economically challenged (17). Moreover, the demand for privately purchased health insurance remains low (18, 19).

In addition to the low enrolment rates in PFHI and low demand for private schemes, health insurance metrics also show fragmented coverage across states and districts of India (16, 19, 20). Enrolments for RSBY varied from 14% among eligible households in Delhi (21) to 68% in Karnataka (22). Studies carried out in Maharashtra have noted variations across and within districts (15, 23). Examining contextual factors will improve our understanding of health insurance uptake, as maximum coverage under PFHI comes from state health schemes that vary in their depth, service coverage and implementation (19).

Thus, assessing geographic variations and enrolment level trends is necessary to successfully monitor health insurance interventions. Existing evidence has been based either on older datasets or primary unrepresentative surveys. To address this data gap, our nationally representative study provides updated health insurance coverage estimates and their socioeconomic and geographic patterns, and uses multilevel modelling to decompose its variation by communities, districts, and states. We also examine the association between coverage and household factors, while accounting for the clustering of populations at multiple levels of geographies.

## Methods

### Dataset

We have used cross-sectional data from the National Family Health Survey Round 5 (NFHS-5), conducted in 2020–21, in 707 districts of India (24). The survey used a two-stage stratified sampling design and selected the primary sampling units (PSUs) by probability proportional to size (PPS). In the first stage, villages and census enumeration blocks were selected as PSUs in rural and urban areas. In all, 30,456 PSUs were selected across 707 districts in India, using the 2011 census as the sampling frame. A “cluster” is a group of adjacent households that serves as a primary sampling unit if the number of households in a given village /census enumeration block is more than 300. Thus, a cluster either represents a village/census enumeration block or a part of it. Throughout the study, we have used clusters as our geographic reference unit for PSU and will hereafter referred to them as “communities.” In the second stage, after a complete mapping and household listing of the selected PSUs, 22 households were selected randomly. Overall, 664,972 households were selected for the sample, of which 653,144 were occupied, and 636,699 (160,138 urban and 476,561 rural) were successfully interviewed (with a response rate of 98%). Household response rates were high in all states except Chandigarh (88%) and Madhya Pradesh (93.7%). Further details of sampling, coverage and data collection are available in the national report (24).

### Health insurance

The study’s primary outcome is “any insurance,” which refers to households where some health insurance covers at least one individual. The respondent (usually the household head) was asked, “Is any usual member of this household covered by a health scheme or health insurance?” Those who responded affirmatively (“Yes”) were then asked, “What type of health scheme or health insurance?” with options such as the employees state insurance scheme (ESIS), central government health scheme (CGHS), state health insurance, Rashtriya Swasthya Bima Yojana (RSBY), community health insurance, other health insurance through an employer, medical reimbursement from an employer, other privately purchased commercial health insurance, and any other form of health insurance that was not covered in options listed above.

Four types of insurance, the government-sponsored Employees’ State Insurance Corporation (ESIS), Central Government Health Scheme (CGHS), health insurance through the employer, and medical reimbursement from the employer, were combined into one variable as “employer-provided health insurance.” As community health insurance coverage was found to be lower than 1%, we have excluded the option in our analysis. Therefore, we have six outcome variables: “any insurance” and five additional types of insurance, namely, ESIS, the state health insurance scheme, RSBY, privately purchased, and “other” health insurance.

At the time of the survey, Ayushman Bharat/Pradhan Mantri Jan Arogya Yojana (PMJAY) was not fully rolled out, and hence its coverage may have been only partially factored in the sample. Given that PMJAY was introduced in 2018, we assume it is included in “other” health insurance.

### Explanatory variables

We have considered the following information in our analysis: wealth index (lowest, low, middle, high, highest), years of education of the household head (illiterate, 1 to 4 years, 5 to 9 years, 10 to 12 years, 12 years or more), caste (scheduled caste, scheduled tribe, other backward castes, general, do not know), place of residence (urban, rural), age of household head (less than 30 years, 30 to 44 years, 45 to 59 years, 60 to 74 years, 75 years or above), religion (Hindu, Muslim, others), household size (four or less, more than four), gender of household head (male, female) and marital status of household head (currently unmarried, currently married). Household wealth was represented by a composite measure of cumulative living standard based on household assets.

### Statistical analysis

We calculated overall health insurance coverage and its types by socioeconomic and demographic correlates of the household. This analysis was bifurcated for rural and urban subsamples. A separate analysis was done for males and females of reproductive ages. We calculated the percentage of health insurance coverage by its types (as noted in Methods) for a subsample of those households where at least one member is covered by some form of health insurance or health scheme. Further, we examined the geographic variation in the health insurance coverage for households across states and Union Territories of India and mapped the district-wise prevalence for the same.

We used random intercept logistic regression to measure the variation attributable to four levels of geographic nesting, i.e., households (level 1), communities (level 2), district (level 3) and state (level 4). The multilevel regression was also used to measure the association of health insurance coverage with major socioeconomic and demographic characteristics of households. The following multilevel regression equation was used to fit the model:


$$\log I\left(\pi_{ijkl}\right)=\beta_{0}+BX_{ijkl}+g_{0l}+f_{0kl}+v_{0jkl}$$

which estimates the probability of a household in local community ‘j’, district ‘k’ and state ‘l’ being covered by health insurance. In this model, $g_{0l},f_{0kl}\;and\;v_{0ijk}$ indicate the residual difference at the state, district, and community levels, respectively. Each set of residuals are assumed to be normally distributed with a mean of 0 and a variance of $\;\sigma_{0g}^{2},\sigma_{0f}^{2}\;and\;\sigma_{0v}^{2}$ , representing between-state, between-district and between-cluster variance, respectively. Household level variance is not directly estimated, as it is assumed to come from a logistic distribution with a fixed variance of π23 or 3.29.

Multilevel modelling was performed in the Stata extension of MLWin 3.0 software via Monte Carlo Markov Chain (MCMC) methods using a Gibbs sampler, with the default prior distribution of Iterated Generalised Least Squares (IGLS) estimation as starting values, a burn-in of 500 cycles and monitoring of 5000 iterations of chains.

## Results

The study used unit data on 608,417 households (Supplementary Table S1), of which 82.6% (527,220) of household heads were males, 35.0% (213,692) were aged 45 to 59 years, and 30.9% (190,572) had 5 to 9 years of education. The households were predominantly rural (67.0%; 456,495), Hindu (83.7%; 470,340), belonged to other backward castes (43.7%; 233,700) and fell in the highest wealth quintile (24.1%, 121,857). The subsamples of “type” of health insurance had a socio-demographic distribution similar to the overall sample.

Of the 608,417 households, 259,543 (41.2%, 95% CI 41.0–41.3%) had at least one person with some form of health insurance (Table 1). Within the covered households, 45,129 (15.5%, 95% CI 15.4–15.7%) had RSBY, 107,796 (47.1%, 95% CI 46.9–47.3%) had state sponsored health insurance schemes, 31,215 (13.2%, 95% CI 13.0–13.3%) had an employer provided health scheme, 6384 (3.3%, 95% CI 3.2–3.3%) had privately purchased health insurance, and 79,187 (25.6%, 95% CI 25.4–25.7%) had “other” forms of health insurance.

**Table 1:** Percentage (%) (and 95% CI) of households with at least one member covered by health insurance across demographic and socioeconomic categories in India, 2020–21.

	Any health insurance	Rashtriya Swashtya Bima Yojana (Central Government sponsored)	State Sponsored health scheme	Employer provided	Privately purchased	Other
								*n* = 259,543
*India*	41.2 [41.0–41.3]	15.5 [15.4–15.7]	47.1 [46.9–47.3]	13.2 [13.0–13.3]	3.3 [3.2–3.3]	25.6 [25.4–25.7]
*Wealth quintile*						
Lowest	35.0 [34.7–35.3]	22.2 [21.8–22.5]	31.9 [31.4–32.3]	5.6 [5.4–5.9]	0.2 [0.1–0.2]	43.1 [42.6–43.5]
Second	41.2 [41.0–41.5]	17.0 [16.7–17.3]	46.4 [45.9–46.8]	7.0 [6.8–7.2]	0.4 [0.3–0.4]	33.1 [32.7–33.5]
Third	44.4 [44.2–44.7]	14.5 [14.2–14.8]	56.5 [56.1–56.9]	9.1 [8.8–9.3]	0.9 [0.8–1]	23.6 [23.3–24.0]
Fourth	43.6 [43.3–43.8]	15.4 [15.1–15.7]	56.2 [55.8–56.6]	12.7 [12.4–12.9]	1.8 [1.7–1.9]	19.1 [18.7–19.4]
Highest	40.9 [40.6–41.2]	11.3 [11.0–11.6]	40.5 [40.0–40.9]	26.5 [26.1–26.8]	10.8 [10.5–11.1]	16.8 [16.5–17.1]
*Years of education (household head)*						
Illiterate	41.2 [41.0–41.5]	14.4 [14.2–14.7]	53.4 [53–53.7]	7.5 [7.3–7.7]	0.8 [0.7–0.8]	27.7 [27.4–28.0]
1 to 4 years	44.8 [44.4–45.2]	21.5 [21.0–22.0]	47.0 [46.4–47.6]	10.0 [9.7–10.4]	1.1 [1.0–1.2]	25.7 [25.2–26.2]
5 to 9 years	41.7 [41.5–42.0]	17.7 [17.4–17.9]	47.9 [47.6–48.3]	10.8 [10.6–11.0]	1.7 [1.6–1.8]	26.8 [26.5–27.1]
10 to 12 years	38.8 [38.5–39.1]	14.3 [14.0–14.6]	43 [42.5–43.4]	17.9 [17.6–18.3]	5.1 [4.9–5.3]	24.2 [23.9–24.6]
12 years or more	40.5 [40.1–40.9]	8.6 [8.3–9.0]	34.8 [34.2–35.4]	30.3 [29.7–30.9]	13.7 [13.3–14.2]	18.3 [17.8–18.8]
*Social category of household head*						
Scheduled caste	42.7 [42.4–43.0]	14.9 [14.6–15.2]	48.9 [48.5–49.3]	11.4 [11.1–11.7]	1.3 [1.2–1.4]	28.2 [27.8–28.5]
Scheduled tribe	46.8 [46.5–47.0]	23.8 [23.5–24.2]	40.7 [40.3–41.1]	10.0 [9.8–10.3]	0.6 [0.6–0.7]	28.8 [28.4–29.2]
Other backward castes	43.0 [42.8–43.2]	14.0 [13.7–14.2]	52.0 [51.7–52.3]	12.5 [12.3–12.7]	2.5 [2.4–2.6]	24.1 [23.8–24.4]
General	34.3 [34.0–34.5]	14.9 [14.6–15.3]	37.2 [36.8–37.7]	18.8 [18.5–19.2]	9.1 [8.8–9.4]	23.9 [23.5–24.2]
Do not know	28.1 [26.7–29.4]	21.1 [18.8–23.4]	37.5 [34.7–40.2]	13.3 [11.4–15.2]	3.4 [2.4–4.5]	27.5 [25.0–30.1]
*Place of residence*						
Urban	38.4 [38.1–38.6]	12.5 [12.3–12.8]	43.2 [42.8–43.6]	22.3 [21.9–22.6]	7.7 [7.5–7.9]	19.5 [19.1–19.8]
Rural	42.5 [42.4–42.7]	16.8 [16.7–17.0]	48.8 [48.6–49.1]	9.1 [9.0–9.2]	1.3 [1.3–1.4]	28.3 [28.1–28.5]
*Age of household head*						
Less than 30	27.8 [27.3–28.2]	14.0 [13.4–14.6]	44.4 [43.5–45.2]	11.3 [10.8–11.9]	2.6 [2.3–2.8]	31.2 [30.4–32.0]
30 to 44	39.7 [39.4–39.9]	14.3 [14.1–14.6]	47.1 [46.7–47.4]	12.5 [12.3–12.8]	3 [2.9–3.1]	27.4 [27.0–27.7]
45 to 59	44.7 [44.5–44.9]	16.0 [15.8–16.2]	47.1 [46.8–47.4]	13.5 [13.2–13.7]	3.4 [3.3–3.5]	25 [24.8–25.3]
60 to 74	42.1 [41.8–42.3]	16.3 [16.0–16.6]	47.7 [47.3–48.1]	13.5 [13.2–13.8]	3.3 [3.2–3.4]	23.9 [23.5–24.2]
75 and above	39.1 [38.5–39.7]	16.7 [16.0–17.3]	46.5 [45.6–47.4]	14.8 [14.2–15.5]	4.5 [4.1–4.9]	21.2 [20.5–22.0]
*Religion*						
Hindu	42.5 [42.4–42.7]	14.9 [14.7–15.0]	47.9 [47.6–48.1]	13.3 [13.1–13.4]	3.2 [3.1–3.3]	25.6 [25.4–25.8]
Muslim	29.5 [29.1–29.9]	20.5 [19.9–21.1]	41 [40.2–41.7]	9.4 [8.9–9.8]	1.7 [1.5–1.9]	30.1 [29.4–30.8]
Others	43.1 [42.7–43.4]	18.4 [18.0–18.9]	44.1 [43.6–44.7]	16.1 [15.6–16.5]	6.6 [6.4–6.9]	18.7 [18.3–19.2]
*Household size*						
Four or less	42.4 [42.2–42.5]	15.2 [15.0–15.4]	49.9 [49.7–50.2]	14.2 [14.1–14.4]	3.6 [3.5–3.7]	21.9 [21.7–22.1]
More than four	39.6 [39.4–39.8]	16.0 [15.8–16.2]	43.1 [42.9–43.4]	11.6 [11.5–11.8]	2.8 [2.7–2.9]	30.7 [30.5–31.0]
*Gender of household head*						
Male	41.4 [41.3–41.5]	15.4 [15.2–15.5]	46.7 [46.5–46.9]	13.4 [13.3–13.6]	3.5 [3.4–3.6]	25.7 [25.5–25.8]
Female	40.0 [39.7–40.3]	16.2 [15.8–16.5]	49.1 [48.6–49.5]	11.7 [11.4–12.0]	2.2 [2.1–2.3]	25.0 [24.6–25.4]
*Marital status of household head*						
Currently unmarried	40.6 [40.3–40.9]	16.5 [16.2–16.9]	49.9 [49.5–50.4]	12.0 [11.7–12.3]	2.7 [2.5–2.8]	23.0 [22.5–23.4]
Currently married	41.3 [41.1–41.4]	15.3 [15.2–15.5]	46.6 [46.4–46.8]	13.4 [13.2–13.5]	3.4 [3.3–3.5]	26.0 [25.9–26.2]

The percentage of households having “any” health insurance for at least one family member was the largest among the third wealth quintile (44.4%, 95% CI 44.2–44.7%), when the household head had an education of 1 to 4 years (44.8%, 95% CI 44.4–45.2%) and, when the household head was aged 45 to 59 years (44.7%, 95% CI, 44.5–44.9%). Rural (v/s urban) households had higher coverage [(42.5%, 95% CI 42.4–42.7%) v/s (38.4%, 95% CI 38.1–38.6%)].

In terms of percentage distribution (Supplementary Table S1), households belonging to the lowest wealth quintile accounted only for 15.0% of overall health insurance, 21.4% of RSBY, 10.2% of state health schemes, 6.5% of employer-provided schemes, 1.1% of privately purchased insurance and 25.3% of “other insurance” schemes. Coverage patterns for rural and urban areas by socio-demographic correlates (Supplementary Tables S2, S3) and states (Supplementary Tables S4–S6) are similar to socio-economic and state wise all India trends. No major difference was found in the coverage trend between males and females of reproductive age groups (Supplementary Tables S7; S8).

**Figure 1:**
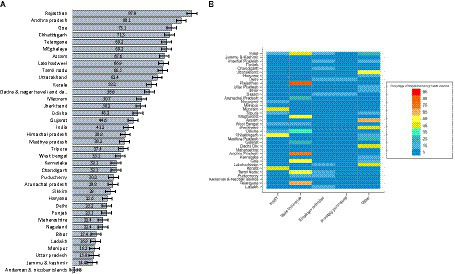
**(A)** Percentage of households with at least one member having any health insurance in states of India, 2019–20 and **(B)** Percentage of households with type of insurance among those who have at least some form of health insurance, India, 2019–20.

Vast state-wise variation in coverage of health insurance in households was noted (Figure 1A), from 87.9% (95% CI 87.6–88.3%) in Rajasthan to 1.8% (95% CI 1.2–2.3%) in Andaman and Nicobar Islands. RSBY coverage varies from 0.1% (95% CI 0.0–0.1%) in Telangana to 54.2% (95% CI 53.5–54.8%) in Chhattisgarh (Supplementary Table S4; Figure 1B). State-sponsored health insurance coverage varies from 0.2% (95% CI 0.1–0.3%) in Tripura to 82.8% (95% CI 82.4–83.2%) in Rajasthan. The coverage of employer-provided health insurance is below 5% in most states, except for certain northern states such as Jammu and Kashmir (9.8%), Himachal Pradesh (10.4%), Punjab (9.1%), Haryana (7.2%), and Uttarakhand (9.0%). Privately purchased health insurance is highest in Delhi at 10.8% (95% CI 10.1–11.4%) and usually lies below 5% for most other states. The geographical comparison of “other” health insurance was not applicable, owing to the staggered and incomplete national rollout of PMJAY (assumed to be the highest component in the “other” category) during the survey timeframe.

**Figure 2:**
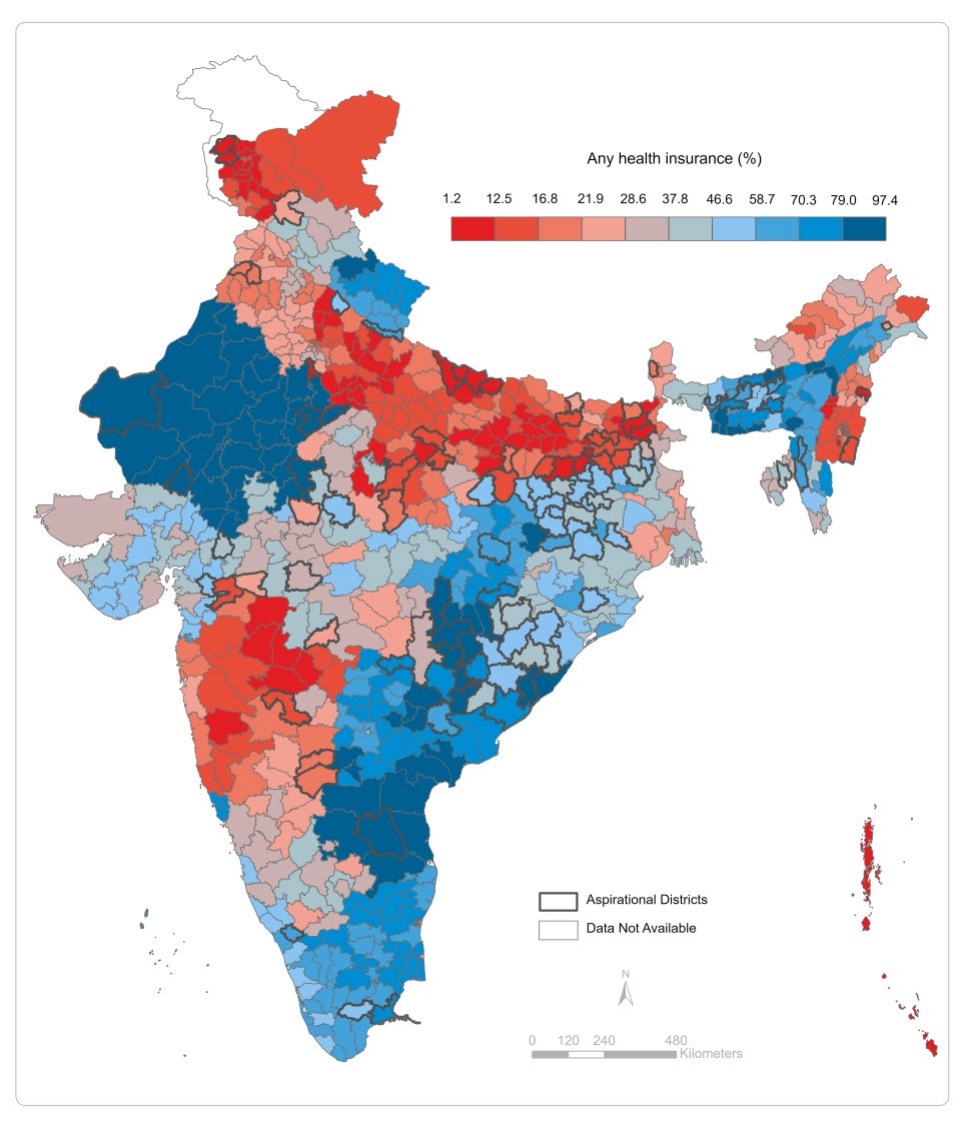
Percentage of households covered by any health insurance across districts of India, 2019–20.

Across districts of India (Figure 2; Supplementary Figures S1–S5), we note that coverage of “any” insurance is higher in the north and north-east (Rajasthan, Uttarakhand, Assam, Meghalaya, and some districts of Tripura), east (some districts of Bihar, Jharkhand, Chhattisgarh, and Odisha), and southeast (Andhra Pradesh, Telangana, and Tamil Nadu). In aspirational districts, coverage of any insurance is usually below 58%, and for RSBY and state-sponsored health insurance, it lies below 2.5% (Supplementary Figure S1).

**Figure 3:**
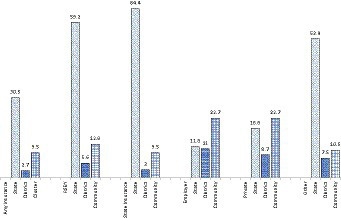
Partitioning Variation (VPC) in insurance coverage among households across multiple geographies, India, NFHS.

The percentage variation in coverage of health insurance accounted for by states is 30.5% for any insurance, 59.2% for RSBY, 64.4% for state-funded health schemes, 11.8% for employer-funded, 18.8% for privately purchased, and 52.9% for “other” health insurance (Figure 3). The variation across districts for overall health insurance coverage and its types lies below 11%. The variation between communities is higher for employer-funded and privately purchased health insurance at 22.7%.

Multilevel logistic estimates show that the odds of “any” insurance, state-funded insurance, employer-provided, and privately purchased health insurance increase with wealth (Table 2). For example, with reference to the lowest wealth quintile, the adjusted odds of privately purchased health insurance are 12.2 (95% CI 10.0–14.6, *p* < 0.001) for the highest quintile. Adjusted odds did not vary significantly with the education of the household head for “any” and “other” insurance. For RSBY and state-sponsored health insurance, the odds declined with the education of the household head, while that of employer-provided and privately purchased insurance increased.

**Table 2:** Adjusted odds ratio (AOR) and confidence intervals (95% CI) for predictors of health insurance coverage by its types, (n=608,417).

	Any insurance		Rashtriya Swasthya Bima Yojana		State insurance		Employer provided		Privately purchased		Other insurance	
	AOR	95% CI	AOR	95% CI	AOR	95% CI	AOR	95% CI	AOR	95% CI	AOR	95% CI
	*n* = 608,417	
*Wealth quintile*												
Lowest	Ref	Ref	Ref	Ref	Ref	Ref	Ref	Ref	Ref	Ref	Ref	Ref
Second	1.2^***^	[1.1–1.2]	1.1^***^	[1–1.1]	1.3^***^	[1.2–1.3]	1.2^***^	[1.1–1.6]	1.5^***^	[1.2–1.9]	1.1^***^	[1.1–1.2]
Third	1.2^***^	[1.2–1.2]	1.1^***^	[1.1–1.2]	1.4^***^	[1.4–1.5]	1.5^***^	[1.4–2.1]	2.7^***^	[2.2–3.3]	1^***^	[1–1.1]
Fourth	1.2^***^	[1.2–1.2]	1.1^***^	[1–1.2]	1.5^***^	[1.4–1.5]	2^***^	[1.9–3.7]	4.4^***^	[3.6–5.3]	0.9^*^	[0.9–1]
Highest	1.3^***^	[1.3–1.4]	0.8^***^	[0.7–0.8]	1.3^***^	[1.2–1.4]	3.5^***^	[3.3–0.8]	12.2^***^	[10.0–14.6]	0.8^***^	[0.8–0.9]
*Years of education (household head)*												
Illiterate	Ref	Ref	Ref	Ref	Ref	Ref	Ref	Ref	Ref	Ref	Ref	Ref
1 to 4 years	1.1^***^	[1.1–1.1]	1^***^	[1–1.1]	1.0^**^	[1–1]	1.1^***^	[1.1–1.2]	1.2^**^	[1–1.4]	1.1^***^	[1–1.1]
5 to 9 years	1.0^***^	[1–1]	1.0^*^	[0.9–1]	0.9	[0.9–1]	1.2^***^	[1.1–1.6]	1.4^***^	[1.2–1.5]	1^***^	[1–1.1]
10 to 12 years	1.0^*^	[1–1]	0.8^***^	[0.8–0.9]	0.8^***^	[0.8–0.9]	1.5^***^	[1.4–2.5]	2^***^	[1.8–2.2]	0.9	[0.9–1]
12 years or more	1.1^***^	[1–1.1]	0.6^***^	[0.6–0.6]	0.7^***^	[0.7–0.8]	2.4^***^	[2.2–1.4]	3.8^***^	[3.3–4.3]	0.8^***^	[0.7–0.8]
*Social category*												
Scheduled caste	Ref	Ref	Ref	Ref	Ref	Ref	Ref	Ref	Ref	Ref	Ref	Ref
Scheduled tribe	0.8^***^	[0.8–0.8]	0.8^***^	[0.8–0.9]	0.9	[0.9–1]	1^**^	[1–0.8]	1	[0.8–1.1]	0.7^***^	[0.7–0.7]
Other backward castes	0.8^***^	[0.8–0.8]	0.8^***^	[0.8–0.8]	0.9^***^	[0.9–0.9]	0.8^***^	[0.8–0.9]	1.3^***^	[1.2–1.5]	0.7^***^	[0.7–0.8]
General	0.7^***^	[0.7–0.7]	0.7^***^	[0.6–0.7]	0.7^***^	[0.7–0.8]	0.9^***^	[0.8–0.8]	1.9^***^	[1.7–2.1]	0.6^***^	[0.6–0.7]
Do not know	0.5^***^	[0.5–0.6]	0.6^***^	[0.5–0.7]	0.6^***^	[0.5–0.6]	0.6^***^	[0.5–1]	1	[0.7–1.5]	0.6^***^	[0.5–0.7]
*Place of residence*												
Urban	Ref	Ref	Ref	Ref	Ref	Ref	Ref	Ref	Ref	Ref	Ref	Ref
Rural	1.2^***^	[1.2–1.3]	1.3^***^	[1.2–1.4]	1.4^***^	[1.3–1.5]	0.8^***^	[0.7–1.1]	0.8^***^	[0.7–0.8]	1	[0.9–1]
*Age (household head)*												
Less than 30	Ref	Ref	Ref	Ref	Ref	Ref	Ref	Ref	Ref	Ref	Ref	Ref
30 to 44	1.6^***^	[1.6–1.7]	1.4^***^	[1.3–1.5]	1.6^***^	[1.5–1.7]	1.3^***^	[1.2–1.7]	1.3^***^	[1.1–1.5]	1.5^***^	[1.5–1.6]
45 to 59	2.1^***^	[2.1–2.2]	1.8^***^	[1.7–1.9]	2.2^***^	[2.1–2.3]	1.6^***^	[1.5–1.8]	1.5^***^	[1.2–1.7]	1.7^***^	[1.6–1.8]
60 to 74	1.9^***^	[1.9–2]	1.6^***^	[1.5–1.8]	2.0^***^	[1.9–2.1]	1.6^***^	[1.5–1.8]	1.4^***^	[1.2–1.6]	1.6^***^	[1.5–1.6]
75 and above	1.7^***^	[1.7–1.8]	1.4^***^	[1.3–1.6]	1.7^***^	[1.6–1.9]	1.7^***^	[1.6–1.3]	1.6^***^	[1.3–2]	1.4^***^	[1.3–1.5]
*Religion*												
Hindu	Ref	Ref	Ref	Ref	Ref	Ref	Ref	Ref	Ref	Ref	Ref	Ref
Muslim	0.7^***^	[0.7–0.8]	0.7^***^	[0.7–0.8]	0.8^***^	[0.8–0.9]	0.6^***^	[0.5–0.9]	0.4^***^	[0.4–0.5]	0.8^***^	[0.8–0.9]
Others	0.9	[0.9–1]	0.8^***^	[0.7–0.9]	1.0^*^	[1–1.1]	0.9^*^	[0.8–1.1]	1.2^***^	[1.1–1.3]	0.9	[0.9–1]
*Household size*												
Four or less	Ref	Ref	Ref	Ref	Ref	Ref	Ref	Ref	Ref	Ref	Ref	Ref
More than four	1.1^***^	[1.1–1.2]	1.1^***^	[1.1–1.2]	1.1^***^	[1.1–1.1]	1	[0.9–0]	1.1^***^	[1–1.1]	1.2^***^	[1.1–1.2]
*Gender (household head)*												
Male	Ref	Ref	Ref	Ref	Ref	Ref	Ref	Ref	Ref	Ref	Ref	Ref
Female	1.0	[0.9–1]	0.9^*^	[0.9–0.9]	0.9^*^	[0.9–1]	1^***^	[0.9–1.1]	0.9	[0.8–1]	1	[0.9–1]
*Marital status (household head)*												
Currently unmarried	Ref	Ref	Ref	Ref	Ref	Ref	Ref	Ref	Ref	Ref	Ref	Ref
Currently married	1.1^***^	[1–1.1]	1**	[1–1.1]	1.0^**^	[1–1]	1.1^***^	[1–1.2]	0.9	[0.8–1]	1.1^***^	[1.1–1.1]
Constant	0.2^***^	[0.2–0.3]	0.02^***^	[0.02–0.03]	0.03^***^	[0.2–0.4]	0.007^***^	[0.005–0.007]	0^***^	[0–0]	0.06^***^	[0.05–0.07]

Level of significance: ^***^p < 0.001; ^**^p < 0.01; ^*^p < 0.05.Note: Estimates based on random intercept four level logistic regression.

## Discussion

Health insurance, which is offered as a demand-side solution to OOPE by proponents of universal health coverage, falls prey to “voltage drops” (25) between the identification of populations eligible for receiving health insurance benefits. The analogy of voltage drops or loss in electricity transmission has been made by Eisenberg (25) with loss in individuals who should benefit from insurance to highlight the downfall in populations at each stage of insurance cascade- eligibility, identification, enrolment and use. The largest loss during transmission on this “insurance cascade” (15, 16) is noted at the stage of enrolment, both for publicly funded and privately purchased insurance. Thus, an analysis of health insurance coverage, which quantifies the level of enrolment in health insurance programs, is a good starting point for diagnosing the gap in health insurance delivery.

We estimate that 41.2% of households in India have at least one member with some form of health insurance, with 33.8% and 29.5% of reproductive age males and females covered. This is significantly higher than estimates from other nationally representative health consumption survey (2018) which found coverage at 14%–19% among individuals (4). The administrative data from Insurance Regulatory and Development Authority of India (IRDAI) shows that about 253 million individuals were covered by health insurance in 2011-12, which was approximately 20% of India’s population. (26) Primary data from three states of Gujarat, Haryana and Uttar Pradesh shows similar coverage (8). Among the poor (lowest and second wealth quintile), we estimated the overall coverage at 35.0–41.2%, which is higher compared to the estimates in similar wealth groups from other health surveys (9.4–14.0%) and primary studies (18–19%) (4, 8). By comparing earlier evidence and prior rounds of the NFHS with our findings, we note an overall increase in health insurance coverage, especially among vulnerable groups. The recently introduced central health insurance scheme, Pradhan Mantri Jan Arogya Yojana (PMJAY), may have contributed to this increase.

While PFHIs such as RSBY and state schemes are higher among the poor and lower castes (vulnerable groups), the percentage distribution of populations covered by these schemes shows that only 27% and 40% of the population falls in the lowest and second wealth quintiles. Including private insurance, which is higher among the well-off, only 34% of the entire population covered by any insurance belong to economically challenged groups. These findings highlights the increasing but still inadequate and socio-economically unequal distribution of health insurance coverage.

We found a significant association of enrolment with caste and religion, but a low association with individual characteristics of the household head, such as education, age, gender, and marital status. Our results are similar to previous evidence from systematic reviews (28) and local studies (27), as well as nationally representative studies from large- scale data (19, 20). Similar to our results, the higher coverage of RSBY in rural areas and private insurance in urban areas has been highlighted previously (19, 20, 29). However, unlike our findings for any insurance coverage, Prinja (8) found higher overall coverage in urban areas (25%) compared to rural areas (18%).

Geographic variation in health insurance coverage has been identified previously by researchers (7, 10, 15, 16), but very few have empirically quantified it (19, 20). Our findings of higher health insurance coverage in the east and southeast India, and some western states such as Rajasthan and Himachal Pradesh, are validated by compiling evidence from primary studies. Karan et al. (13) in their discourse on RSBY, highlight that east and south-eastern states such as Chhattisgarh, Karnataka, Odisha, and Kerala cover 70%–90% of their population under health insurance schemes. Sriram and Khan (17) highlight that compared to the national average of 9.5, 40% of the poorest populations were covered by health schemes in Andhra Pradesh. The lower coverage in western states is reiterated by the findings of Thakur (15) and La Forgia (30). Similar to ours, studies based on previous rounds of the NFHS find that 60% of the population in Andhra Pradesh, Tamil Nadu, Telangana, Chhattisgarh, Arunachal Pradesh, and Tripura are covered by health insurance (19, 20). Additionally, we found that substantial variation in coverage remains attributable to states (i.e., interstate variation) for “any” insurance, RSBY, state-funded schemes, and “other” insurance. Variation is most significant across communities for employer-provided and privately purchased insurances (i.e., across clusters).

We consistently observed that health insurance enrolment inequality (geographic and socioeconomic) is more prominent in urban areas than rural. The pro-rural focus of most PFHIs, while improving health insurance awareness in those areas, has inadvertently caused higher disparity in urban coverage. Ineffective targeting and low awareness among target populations are major reasons for overall coverage inequality. (15, 16, 25, 31) Evidence highlights that regions with a higher awareness of health insurance have higher enrolment rates, as well. For example, states like Chhattisgarh and Karnataka, which have high enrolment, also report high awareness (8,19), whereas states like Maharashtra report low awareness and low coverage (15).

Administrative difficulties such as vast geographical coverage and discrepancies in identifying eligible populations due to lack of accurate data and poor infrastructure at the grass roots level inhibit effective IEC (Information, Education and Communication) and enrolment (8, 15). The lack of effective IEC activities from third party administrators, who are entrusted with IEC responsibilities is one of the primary reasons for low awareness about health insurance (15). Often enrolment campaigns are organized in villages and urban localities on very short notice and without prior preparation (6, 15, 16). Insurance companies that undertake enrolment also find it beneficial to enrol more households over individuals, as reimbursement happens at the household level (15, 16). Further, poor households often do not have proper documentation such as BPL or ADHAAR cards (8, 27). This, coupled with “cream-skimming” (or favouring lower-cost candidates) by administrators, causes the systematic elimination of socioeconomically vulnerable populations from enrolment (7).

Besides the structural factors, social capital plays an essential role in determining enrolment (and awareness) (10). Inadequate knowledge about how health insurance works, which is termed as “understanding failure” (32, 33) and a lack of “demonstration effect” (34) by others who have benefited from insurance in the locality (due to an overall lack of coverage in a region) discourage enrolment among lower socioeconomic groups. Further, political landscape and will that differs by geographic region also impact the effectiveness of health insurance outreach and uptake (35). These factors reiterate the relevance of geographical contexts in determining awareness and overall enrolment in health insurance schemes. Given these realities, our findings on geographical inequality and district-wise enrolment rates are essential for policymakers working to increase health insurance uptake in their communities, especially through place-based interventions for the success of PMJAY.

Our study has several limitations. First, our enrolment rates are not limited to the “eligible” but cover the entire population. The lack of information on community awareness of and eligibility for health insurance prevents understating enrolment in the “insurance cascade” i.e. we do not understand how many of the ‘eligible’ population is not enrolled and just know what the overall enrolment is instead. Secondly, we provide estimates only at the household level and thus, do not estimate overall population coverage. Lastly, the lack of explicit information on PMJAY prevents the generalizability of results for “other” insurance, a significant component of overall coverage.

## Conclusion

In the past 2 decades, health insurance coverage in India has improved among both the overall population and socioeconomically vulnerable groups, yet it remains inadequate. To date, nearly 60% of families do not have a single member covered by any health insurance scheme. The existing coverage is fragmented, with significant rural/urban and geographic variation primarily attributable to states and communities. Individual characteristics of the household head have little impact on overall coverage and it is mainly determined by place of residence, caste, and economic status. It is essential to consider these disparities in order to successfully implement PMJAY with a rigorous focus on place-based interventions, to fill the state and community level gaps in coverage.

## Data availability statement

Publicly available datasets were analyzed in this study. This data can be found at: https://dhsprogram.com/data/dataset/India_Standard-DHS_2020.cfm?flag=0.

## Author contributions

RK and SS conceptualization and design and overall supervision. MA: data acquisition and analysis and writing of the manuscript. MA, SR, RK, and SS: data interpretation and critical revisions. All authors contributed to the article and approved the submitted version.

## Funding

This study was supported by a grant from the Bill & Melinda Gates Foundation INV-002992.

## Conflict of interest

The authors declare that the research was conducted in the absence of any commercial or financial relationships that could be construed as a potential conflict of interest.

## Publisher’s note

All claims expressed in this article are solely those of the authors and do not necessarily represent those of their affiliated organizations, or those of the publisher, the editors and the reviewers. Any product that may be evaluated in this article, or claim that may be made by its manufacturer, is not guaranteed or endorsed by the publisher.

## Supplementary material

The Supplementary material for this article can be found online at: https://www.frontiersin.org/articles/10.3389/fpubh.2023.1160088/full#supplementary-material
