# Supplementary material for: Socioeconomic and geographic variation in coverage of health insurance across India
Source: Front Public Health. 2023 Jul 10;11:1160088. doi: 10.3389/fpubh.2023.1160088 (PMC10365087; doi:10.3389/fpubh.2023.1160088)
Supplement: Supplementary file 1 [file Data_Sheet_1.docx]

Supplementary analysis

**Title:**

Socioeconomic and geographic variation in coverage of health insurance across India

**Table S1 Distribution of study sample (total and by type of insurance), India, 2020-21**

|  | **Total** | | **Any insurance** | | **RSBY** | | **State Insurance** | | **Employer** | | **Private** | | **Other** | |
| --- | --- | --- | --- | --- | --- | --- | --- | --- | --- | --- | --- | --- | --- | --- |
|  | **n** | **%** | **n** | **%** | **n** | **%** | **n** | **%** | **n** | **%** | **n** | **%** | **n** | **%** |
| **India** | 608417 | 100 | 259,543 | 100 | 45129 | 100 | 107,796 | 100 | 31,215 | 100 | 6348 | 100 | 79,187 | 100 |
|  |  |  |  |  |  |  |  |  |  |  |  |  |  |  |
| **Gender of household head** |  |  |  |  |  |  |  |  |  |  |  |  |  |  |
| Male | 527,220 | 82.6 | 214,319 | 82.9 | 37,388 | 82.2 | 87,899 | 82.2 | 26,520 | 84.7 | 5,551 | 88.4 | 65,559 | 83.3 |
| Female | 109,463 | 17.5 | 45,223 | 17.1 | 7,741 | 17.8 | 19,897 | 17.8 | 4,695 | 15.3 | 797 | 11.6 | 13,627 | 16.8 |
|  |  |  |  |  |  |  |  |  |  |  |  |  |  |  |
| **Age of household head** |  |  |  |  |  |  |  |  |  |  |  |  |  |  |
| Less than 30 | 41,464 | 6.6 | 13,080 | 4.5 | 2,208 | 4.1 | 5,472 | 4.2 | 1,312 | 3.9 | 232 | 3.5 | 4,212 | 5.5 |
| 30 to 44 | 187,435 | 30.5 | 77,795 | 29.4 | 12,812 | 27.1 | 32,486 | 29.4 | 8,917 | 28.0 | 1,706 | 27.0 | 24,609 | 31.4 |
| 45 to 59 | 213,692 | 35.0 | 97,602 | 38.0 | 17,419 | 39.2 | 40,296 | 38.0 | 12,103 | 38.9 | 2,482 | 39.5 | 29,454 | 37.2 |
| 60 to 74 | 138,645 | 23.4 | 59,992 | 24.0 | 10,622 | 25.1 | 25,064 | 24.3 | 7,419 | 24.6 | 1,542 | 24.2 | 17,836 | 22.4 |
| 75 and above | 27,181 | 4.4 | 11,074 | 4.2 | 2,068 | 4.5 | 4,478 | 4.2 | 1,464 | 4.8 | 386 | 5.8 | 3,076 | 3.5 |
|  |  |  |  |  |  |  |  |  |  |  |  |  |  |  |
| **Wealth quintile** |  |  |  |  |  |  |  |  |  |  |  |  |  |  |
| Lowest | 121,258 | 17.7 | 47,151 | 15.0 | 11,331 | 21.4 | 13,882 | 10.2 | 2,527 | 6.5 | 86 | 1.1 | 20,607 | 25.3 |
| Second | 121,073 | 17.7 | 52,700 | 17.7 | 9,574 | 19.4 | 21,322 | 17.4 | 3,579 | 9.4 | 190 | 2.3 | 19,729 | 22.9 |
| Third | 122,281 | 19.3 | 55,655 | 20.9 | 9,144 | 19.5 | 27,297 | 25.0 | 4,920 | 14.4 | 477 | 5.9 | 15,926 | 19.3 |
| Fourth | 121,948 | 21.3 | 53,978 | 22.5 | 8,961 | 22.3 | 26,534 | 26.8 | 6,999 | 21.7 | 962 | 12.5 | 12,907 | 16.8 |
| Highest | 121,857 | 24.1 | 50,059 | 23.9 | 6,119 | 17.4 | 18,761 | 20.6 | 13,190 | 48.1 | 4,633 | 78.2 | 10,018 | 15.7 |
|  |  |  |  |  |  |  |  |  |  |  |  |  |  |  |
| **Education of household head** |  |  |  |  |  |  |  |  |  |  |  |  |  |  |
| Illiterate | 183,720 | 29.1 | 77,691 | 29.1 | 12,728 | 27.1 | 37,103 | 33.0 | 5,572 | 16.6 | 501 | 7.1 | 24,226 | 31.6 |
| 1 to 4 years | 56,913 | 9.4 | 26,903 | 10.2 | 6,267 | 14.2 | 10,923 | 10.2 | 2,332 | 7.8 | 267 | 3.6 | 8,356 | 10.3 |
| 5 to 9 years | 190,572 | 30.9 | 82,784 | 31.3 | 16,189 | 35.6 | 33,406 | 31.9 | 8,325 | 25.7 | 1,201 | 16.1 | 27,005 | 32.8 |
| 10 to 12 years | 119,919 | 19.8 | 48,540 | 18.7 | 7,506 | 17.2 | 18,424 | 17.0 | 8,283 | 25.4 | 1,889 | 29.1 | 14,315 | 17.7 |
| 12 years or more | 57,293 | 10.8 | 23,625 | 10.7 | 2,439 | 5.9 | 7,940 | 7.9 | 6,703 | 24.5 | 2,490 | 44.2 | 5,285 | 7.7 |
|  |  |  |  |  |  |  |  |  |  |  |  |  |  |  |
| **Social category** |  |  |  |  |  |  |  |  |  |  |  |  |  |  |
| Scheduled caste | 122,881 | 22.7 | 53,461 | 23.6 | 8,263 | 22.6 | 23137 | 24.5 | 5,801 | 20.4 | 654 | 9.8 | 17,720 | 25.9 |
| Scheduled tribe | 123,286 | 10.0 | 57,504 | 11.3 | 16,019 | 17.4 | 21432 | 9.8 | 5,734 | 8.6 | 441 | 2.3 | 15,643 | 12.8 |
| Other backward castes | 233,700 | 43.7 | 103,390 | 45.6 | 15,095 | 41.0 | 48924 | 50.3 | 11,094 | 43.3 | 2,097 | 35.1 | 30,794 | 43.0 |
| General | 124,273 | 22.7 | 44,003 | 18.9 | 5,564 | 18.2 | 13851 | 15.0 | 8,431 | 27.1 | 3,119 | 52.2 | 14,645 | 17.7 |
| Don’t know | 4,277 | 0.9 | 1,185 | 0.6 | 188 | 0.8 | 452 | 0.5 | 155 | 0.6 | 37 | 0.7 | 385 | 0.7 |
|  |  |  |  |  |  |  |  |  |  |  |  |  |  |  |
| **Religion** |  |  |  |  |  |  |  |  |  |  |  |  |  |  |
| Hindu | 470,340 | 83.7 | 211,520 | 86.5 | 34,618 | 82.9 | 88,974 | 87.8 | 24,422 | 87.3 | 5,069 | 83.9 | 67,434 | 86.7 |
| Muslim | 58,309 | 10.6 | 16,688 | 7.6 | 2,420 | 10.0 | 6,006 | 6.6 | 2,207 | 5.4 | 340 | 4.1 | 6,135 | 9.0 |
| Others | 79,768 | 5.7 | 31,335 | 6.0 | 8,091 | 7.1 | 12,816 | 5.6 | 4,586 | 7.3 | 939 | 12.0 | 5,618 | 4.4 |
|  |  |  |  |  |  |  |  |  |  |  |  |  |  |  |
| **Household size** |  |  |  |  |  |  |  |  |  |  |  |  |  |  |
| Four or less | 356,537 | 56.9 | 146,338 | 58.4 | 25,453 | 57.1 | 63,335 | 61.9 | 18,587 | 63.2 | 3,871 | 64.8 | 40,964 | 50.0 |
| More than four | 280,162 | 43.1 | 113,205 | 41.6 | 19,676 | 42.9 | 44,461 | 38.1 | 12,628 | 36.8 | 2,477 | 35.2 | 38,223 | 50.0 |
|  |  |  |  |  |  |  |  |  |  |  |  |  |  |  |
| **Marital status of household head** |  |  |  |  |  |  |  |  |  |  |  |  |  |  |
| Currently unmarried | 99,161 | 16.1 | 41,809 | 15.9 | 7,859 | 16.9 | 17,927 | 16.8 | 4,485 | 14.5 | 907 | 12.9 | 12,027 | 14.3 |
| Currently married | 509,256 | 83.9 | 217,734 | 84.1 | 37,270 | 83.1 | 89,869 | 83.2 | 26,730 | 85.5 | 5,441 | 87.1 | 67,160 | 85.8 |
|  |  |  |  |  |  |  |  |  |  |  |  |  |  |  |
| **Place of residence** |  |  |  |  |  |  |  |  |  |  |  |  |  |  |
| Urban | 151,922 | 32.94 | 60,827 | 30.73 | 8,746 | 24.84 | 23,863 | 28 | 12,811 | 51.94 | 4,013 | 71.67 | 14,155 | 23 |
| Rural | 456,495 | 67.06 | 198,716 | 69.27 | 36,383 | 75.16 | 83,933 | 71.82 | 18,404 | 48.06 | 2,335 | 28.33 | 65,032 | 77 |

# Table S2 Percentage of households with at least one member covered by health insurance (and its types) across demographic and socioeconomic categories in urban India, 2020-21

|  | **Any health insurance** | **RSBY** | **State** | **Employer** | **Privately purchased** | **Other** |
| --- | --- | --- | --- | --- | --- | --- |
|  |  |  | | | | |
| India | 38.4  [38.1-38.6] | 12.5  [12.3-12.8] | 43.2  [42.8-43.6] | 22.3  [21.9-22.6] | 7.7  [7.5-7.9] | 19.5  [19.1-19.8] |
|  |  |  |  |  |  |  |
| Gender of household head |  |  |  |  |  |  |
| Male | 38.6  [38.3-38.8] | 12.1  [11.8-12.4] | 42.5  [42-42.9] | 23.0  [22.7-23.4] | 8.2  [8.p-8.5] | 19.5  [19.2-19.9] |
| Female | 37.6  [37-38.2] | 14.8  [14.1-15.5] | 46.7  [45.8-47.7] | 18.5[17.7-19.2] | 5.1  [4.7-5.5] | 19.2  [18.5-20] |
|  |  |  |  |  |  |  |
| Marital status of household head |  |  |  |  |  |  |
| Currently unmarried | 36.9  [36.3-37.5] | 14.3  [13.6-14.9] | 46.3  [45.4-47.3] | 19.2  [18.4-19.9] | 5.9  [5.4-6.3] | 18.5  [17.8-19.3] |
| Currently married | 38.7  [38.4-39] | 12.2  [11.9-12.5] | 42.6  [42.1-43] | 22.9  [22.5-23.3] | 8.1  [7.8-8.3] | 19.6  [19.3-20] |
|  |  |  |  |  |  |  |
| Years of education (household head) |  |  |  |  |  |  |
| Illiterate | 36.6  [36-37.1] | 11.5  [10.8-12.1] | 56.1  [55.2-57.1] | 11.7  [11.0-12.3] | 1.6  [1.3-1.8] | 23.6  [22.8-24.4] |
| 1 to 4 years | 39.1  [38.2-40] | 20.1  [18.9-21.2] | 47.6  [46.2-49.1] | 14.5  [13.4-15.5] | 2.2  [1.7-2.6] | 20.8  [19.6-22] |
| 5 to 9 years | 38.1  [37.6-38.5] | 17.1  [16.6-17.7] | 48.1  [47.3-48.8] | 16.3  [15.7-16.8] | 3.3  [3-3.6] | 20.6  [20.0-21.2] |
| 10 to 12 years | 37.2  [36.7-37.6] | 11.8  [11.3-12.3] | 39.8  [39-40.6] | 25.4  [24.7-26.1] | 9.0  [8.5-9.4] | 19.3  [18.7-19.9] |
| 12 years or more | 41.7  [41.1-42.3] | 5.8  [5.4-6.2] | 29.7  [28.9-30.5] | 36.9  [36.1-37.7] | 18.3  [17.6-18.9] | 14.7  [14.1-15.3] |
|  |  |  |  |  |  |  |
| Age of household head |  |  |  |  |  |  |
| Less than 30 | 24.2  [23.3-25.1] | 10.4  [9.2-11.7] | 41.2  [39.2-43.2] | 23.4  [21.7-25.1] | 6.2  [5.2-7.2] | 22.7  [21.0-24.4] |
| 30 to 44 | 35.5  [35.1-35.9] | 11.6  [11.1-12.1] | 44.0  [43.2-44.7] | 21.3  [20.7-22.0] | 7.1  [6.7-7.5] | 20.7  [20.1-21.3] |
| 45 to 59 | 41.5  [41-41.9] | 12.6  [12.2-13] | 43.6  [43.0-44.2] | 22.2  [21.7-22.7] | 7.7  [7.3-8.0] | 19.5  [19.0-20.0] |
| 60 to 74 | 40.7  [40.2-41.2] | 13.5  [12.9-14] | 42.7  [41.8-43.5] | 22.9  [22.2-23.6] | 8.1  [7.7-8.6] | 18.2  [17.6-18.8] |
| 75 and above | 37.8  [36.7-39] | 14.0  [12.7-15.3] | 39.3  [37.5-41.1] | 24.4  [22.9-26.0] | 10.8  [9.6-11.9] | 15.9  [14.5-17.2] |
|  |  |  |  |  |  |  |
| Religion of household head |  |  |  |  |  |  |
| Hindu | 40.0  [39.7-40.3] | 11.8  [11.5-12.1] | 43.1  [42.7-43.6] | 23.3  [23.0-23.7] | 7.9  [7.6-8.1] | 19.5  [19.1-19.8] |
| Muslim | 28.6  [28-29.2] | 17.5  [16.6-18.4] | 45.6  [44.4-46.8] | 12.5  [11.7-13.2] | 2.9  [2.5-3.3] | 24.5  [23.5-25.5] |
| Others | 40.0  [39.3-40.7] | 14.5  [13.6-15.3] | 40.2  [39.1-41.4] | 24.7  [23.7-25.7] | 13.8  [12.9-14.6] | 11.1  [10.3-11.8] |
|  |  |  |  |  |  |  |
| Social category |  |  |  |  |  |  |
| Scheduled caste | 36.9  [36.3-37.5] | 14.8  [14.1-15.5] | 47.5  [46.6-48.5] | 18.4  [17.6-19.1] | 3.1  [2.8-3.5] | 21.3  [20.5-22.0] |
| Scheduled tribe | 38.9  [38.1-39.6] | 17.8  [16.9-18.7] | 36.7  [35.5-37.8] | 23.6  [22.6-24.6] | 3.0  [2.6-3.4] | 22.8  [21.8-23.8] |
| Other backward castes | 42.9  [42.5-43.2] | 12.3  [11.9-12.7] | 49.9  [49.3-50.5] | 20.3  [19.8-20.8] | 4.9  [4.7-5.2] | 18.5  [18.0-18.9] |
| General | 33.4  [33-33.9] | 10.5  [10-11] | 29.2  [28.5-29.9] | 28.4  [27.7-29.1] | 16.7  [16.1-17.3] | 19.3  [18.6-19.9] |
| Don’t know | 23.5  [21.3-25.7] | 13.1  [9.5-16.7] | 33.4  [28.4-38.4] | 21.6  [17.3-26.0] | 8.7  [5.7-11.7] | 26.7  [22.1-31.4] |
|  |  |  |  |  |  |  |
| Household size |  |  |  |  |  |  |
| Four or less | 39.4  [39.1-39.7] | 11.8  [11.5-12.1] | 44.9  [44.4-45.5] | 23.8  [23.3-24.2] | 8.2  [7.9-8.5] | 16.6  [16.2-17.0] |
| More than four | 36.8  [36.4-37.2] | 13.8  [13.4-14.3] | 40.1  [39.5-40.8] | 19.7  [19.2-20.2] | 6.8  [6.5-7.2] | 24.4[23.8-24.9] |
|  |  |  |  |  |  |  |
| Wealth quintile |  |  |  |  |  |  |
| Lowest | 28.4  [27.1-29.7] | 22.2  [20.2-24.3] | 36.4  [33.9-38.8] | 11.3  [9.7-12.9] | 0.3  [0-0.7] | 33.2  [30.9-35.6] |
| Second | 33.7  [32.7-34.7] | 20.3  [18.9-21.6] | 44.6  [42.9-46.3] | 10.1  [9.0-11.1] | 0.5  [0.3-0.8] | 29.1  [27.6-30.7] |
| Third | 35.6  [34.9-36.2] | 15.2  [14.4-16] | 51.9  [50.8-52.9] | 12.0  [11.3-12.7] | 1.2  [1.0-1.5] | 24.2  [23.2-25.1] |
| Fourth | 37.9  [37.4-38.4] | 15.8  [15.2-16.3] | 51.3  [50.5-52.1] | 15.4  [14.8-15.9] | 2.1  [1.9-2.3] | 20.5  [19.9-21.1] |
| Highest | 40.4  [40-40.7] | 9.4  [9.0-9.7] | 37.6  [37.1-38.1] | 29.3  [28.8-29.8] | 12.7  [12.3-13.0] | 16.6  [16.2-17.0] |

# Table S3 Percentage of households with at least one member covered by health insurance (and its types) across demographic and socioeconomic categories in rural India, 2020-21

|  | **Any health insurance** | **RSBY** | **State** | **Employer** | **Privately purchased** | **Other** |
| --- | --- | --- | --- | --- | --- | --- |
|  |  | n= | | | | |
| India | 42.5  [42.4-42.7] | 16.8  [16.7-17.0] | 48.8  [48.6-49.1] | 9.1  [9.0-9.2] | 1.3  [1.3-1.4] | 28.3  [28.1-28.5] |
|  |  |  |  |  |  |  |
| Gender of household head |  |  |  |  |  |  |
| Male | 42.8  [42.7-43] | 16.9  [16.7-17.0] | 48.6  [48.3-48.8] | 9.2  [9.0-9.3] | 1.4  [1.3-1.4] | 28.4  [28.2-28.6] |
| Female | 41.2  [40.8-41.5] | 16.8  [16.4-17.2] | 50.1  [49.5-50.6] | 8.8  [8.5-9.1] | 0.9  [0.8-1.1] | 27.6  [27.1-28.0] |
|  |  |  |  |  |  |  |
| Marital status of household head |  |  |  |  |  |  |
| Currently unmarried | 42.7  [42.3-43] | 17.6  [17.2-18.0] | 51.7  [51.1-52.2] | 8.6  [8.3-8.9] | 1.1  [1.0-1.2] | 25.1  [24.6-25.5] |
| Currently married | 42.5  [42.3-42.7] | 16.7  [16.5-16.9] | 48.3  [48.1-48.6] | 9.2  [9.1-9.3] | 1.3  [1.3-1.4] | 28.9  [28.6-29.1] |
|  |  |  |  |  |  |  |
| Years of education (household head) |  |  |  |  |  |  |
| Illiterate | 42.4  [42.1-42.6] | 15.1  [14.8-15.3] | 52.8  [52.4-53.2] | 6.6  [6.4-6.8] | 0.6  [0.5-0.6] | 28.5  [28.2-28.9] |
| 1 to 4 years | 46.7  [46.2-47.2] | 21.9  [21.4-22.4] | 46.8  [46.1-47.4] | 8.8  [8.4-9.2] | 0.8  [0.7-0.9] | 27.1  [26.5-27.7] |
| 5 to 9 years | 43.4  [43.1-43.6] | 17.9  [17.6-18.2] | 47.9  [47.5-48.3] | 8.6  [8.4-8.8] | 1.0  [0.9-1.1] | 29.2  [28.9-29.6] |
| 10 to 12 years | 40.1  [39.8-40.4] | 16.1  [15.7-16.5] | 45.2  [44.7-45.8] | 12.6  [12.2-13.0] | 2.4  [2.2-2.6] | 27.8  [27.3-28.2] |
| 12 years or more | 38.5  [37.9-39] | 13.6  [12.9-14.2] | 43.9  [43.0-44.8] | 18.5  [17.8-19.2] | 5.6  [5.1-6.0] | 24.8  [24.0-25.7] |
|  |  |  |  |  |  |  |
| Age of household head |  |  |  |  |  |  |
| Less than 30 | 29.1  [28.6-29.6] | 15.1  [14.4-15.8] | 45.4  [44.4-46.3] | 7.7  [7.2-8.2] | 1.5  [1.2-1.7] | 33.7  [32.8-34.6] |
| 30 to 44 | 41.6  [41.4-41.9] | 15.4  [15.1-15.7] | 48.4  [48.0-48.8] | 8.9  [8.7-9.1] | 1.3  [1.2-1.4] | 30.1  [29.7-30.4] |
| 45 to 59 | 46.4  [46.2-46.6] | 17.6  [17.4-17.9] | 48.8  [48.4-49.2] | 9.3  [9.1-9.5] | 1.4  [1.3-1.5] | 27.7  [27.4-28.0] |
| 60 to 74 | 42.8  [42.5-43.1] | 17.6  [17.2-17.9] | 50.1  [49.6-50.5] | 9.1  [8.9-9.4] | 1.1  [1.0-1.2] | 26.5  [26.1-26.9] |
| 75 and above | 39.7  [39.1-40.4] | 17.9  [17.1-18.7] | 49.9  [48.8-51.0] | 10.3  [9.6-10.9] | 1.5  [1.3-1.8] | 23.8  [22.8-24.7] |
|  |  |  |  |  |  |  |
| Religion of household head |  |  |  |  |  |  |
| Hindu | 43.7  [43.5-43.8] | 16.2  [16.0-16.4] | 49.8  [49.6-50.1] | 9.1  [8.9-9.2] | 1.2  [1.2-1.3] | 28.2  [28.0-28.4] |
| Muslim | 30.2  [29.7-30.7] | 22.8  [21.9-23.6] | 37.5  [36.5-38.4] | 7.0  [6.5-7.6] | 0.9  [0.7-1.0] | 34.4  [33.4-35.3] |
| Others | 44.8  [44.4-45.1] | 20.4  [19.9-20.9] | 46.1  [45.5-46.7] | 11.8  [11.4-12.2] | 3.1  [2.9-3.4] | 22.5  [22.0-23.0] |
|  |  |  |  |  |  |  |
| Social category |  |  |  |  |  |  |
| Scheduled caste | 44.9  [44.6-45.3] | 14.9  [14.6-15.3] | 49.4  [48.9-49.8] | 9.1  [8.9-9.4] | 0.8  [0.7-0.8] | 30.4  [29.9-30.8] |
| Scheduled tribe | 48.1  [47.8-48.4] | 24.6  [24.2-25.0] | 41.3  [40.9-41.7] | 8.2  [7.9-8.4] | 0.3  [0.3-0.4] | 29.6  [29.2-30.0] |
| Other backward castes | 43.0  [42.8-43.2] | 14.8  [14.5-15.0] | 53.0  [52.7-53.4] | 8.6  [8.4-8.8] | 1.3  [1.2-1.4] | 26.9  [26.6-27.2] |
| General | 35.0  [34.6-35.3] | 18.4  [18.0-18.9] | 43.5  [42.9-44.1] | 11.3  [11.0-11.7] | 3.2  [3.0-3.4] | 27.4  [26.9-27.9] |
| Don’t know | 31.2  [29.5-32.9] | 25.2  [22.3-28.2] | 39.6  [36.3-42.9] | 9.0  [7.1-10.9] | 0.7  [0.1-1.3] | 27.9  [24.9-31.0] |
|  |  |  |  |  |  |  |
| Household size |  |  |  |  |  |  |
| Four or less | 44.1  [43.9-44.3] | 16.9  [16.6-17.1] | 52.4  [52.1-52.7] | 9.5  [9.3-9.6] | 1.4  [1.3-1.4] | 24.5  [24.3-24.8] |
| More than four | 40.7  [40.5-40.9] | 16.8  [16.6-17.1] | 44.2  [43.9-44.6] | 8.7  [8.5-8.8] | 1.3  [1.2-1.3] | 33.1  [32.8-33.4] |
|  |  |  |  |  |  |  |
| Wealth quintile |  |  |  |  |  |  |
| Lowest | 35.4  [35.1-35.6] | 22.2  [21.8-22.5] | 31.7  [31.2-32.1] | 5.4  [5.2-5.6] | 0 | 43.5  [43.0-43.9] |
| Second | 42.1  [41.8-42.4] | 16.7  [16.4-17.0] | 46.5  [46.1-47.0] | 6.7  [6.5-6.9] | 0.4  [0.3-0.4] | 33.5  [33.1-33.9] |
| Third | 46.9  [46.6-47.2] | 14.3  [14.0-14.7] | 57.5  [57.1-57.9] | 8.5  [8.2-8.7] | 0.8  [0.7-0.9] | 23.5  [23.1-23.9] |
| Fourth | 47.5  [47.2-47.9] | 15.2  [14.8-15.5] | 59.0  [58.5-59.5] | 11.1  [10.8-11.5] | 1.6  [1.5-1.7] | 18.3  [17.9-18.7] |
| Highest | 42.2  [41.8-42.7] | 15.9  [15.4-16.4] | 47.3  [46.6-48.1] | 19.7  [19.2-20.3] | 6.3  [6.0-6.7] | 17.2  [16.7-17.8] |

# Table S4 Percentage of households with at least one member covered by health insurance (and its types) across states in India, 2020-21

| **States** | **% (95% CI)** | | | | | |
| --- | --- | --- | --- | --- | --- | --- |
|  | **Any health insurance** | **RSBY** | **State** | **Employer** | **Privately purchased** | **Other** |
|  |  |  |  |  |  |  |
| India | 41.2  [41-41.3] | 15.5  [15.4-15.7] | 47.1  [46.9-47.3] | 13.2  [13-13.3] | 3.3  [3.2-3.3] | 25.6  [25.4-25.7] |
| Jammu & Kashmir | 14.4  [13.8-15] | 0.4  [0.3-0.5] | 2.1  [1.9-2.3] | 9.8  [9.3-10.3] | 0.8  [0.6-0.9] | 1.6  [1.4-1.8] |
| Himachal Pradesh | 38.8  [37.9-39.7] | 4.7  [4.3-5.2] | 7.9  [7.4-8.4] | 10.4  [9.8-11.0] | 0.8  [0.6-1] | 15.6  [14.9-16.3] |
| Punjab | 25.1  [24.5-25.7] | 3.6  [3.3-3.9] | 6.7  [6.4-7.1] | 9.1  [8.7-9.5] | 2.2  [2-2.5] | 3.6  [3.3-3.9] |
| Chandigarh | 32.1  [28.7-35.4] | 1.8  [0.9-2.8] | 6.8  [5-8.6] | 11.9  [9.6-14.2] | 9.5  [7.4-11.6] | 2.4  [1.3-3.5] |
| Uttarakhand | 62.4  [61.6-63.3] | 2.1  [1.8-2.3] | 2.7  [2.4-3] | 9.0  [8.5-9.5] | 1.1  [0.9-1.3] | 50.7  [49.8-51.6] |
| Haryana | 25.6  [25-26.3] | 3.7  [3.4-3.9] | 1.5  [1.3-1.6] | 7.2  [6.9-7.6] | 2.9  [2.6-3.1] | 10.5  [10.1-11] |
| Delhi | 25.2  [24.3-26] | 0.3  [0.1-0.4] | 1.3  [1.1-1.6] | 12.3  [11.6-12.9] | 10.8  [10.1-11.4] | 1.0  [0.7-1.2] |
| Rajasthan | 87.9  [87.6-88.3] | 3.1  [2.9-3.3] | 82.8  [82.4-83.2] | 2.8  [2.7-3.0] | 1.2  [1.1-1.3] | 0.6  [0.5-0.6] |
| Uttar Pradesh | 15.8  [15.5-16.1] | 1.0  [0.9-1.1] | 0.2  [0.2-0.3] | 1.6  [1.5-1.7] | 0.4  [0.4-0.5] | 12.5  [12.2-12.7] |
| Bihar | 17.4  [17-17.7] | 3.1  [2.9-3.3] | 0.2  [0.1-0.2] | 1.7  [1.5-1.8] | 0.1  [0.1-0.2] | 12.2  [11.8-12.5] |
| Sikkim | 28  [26.5-29.5] | 0.6  [0.3-0.9] | 5.9  [5.1-6.7] | 10.6  [9.6-11.6] | 10.0  [9-11] | 0.9  [0.6-1.2] |
| Arunachal Pradesh | 29.8  [29.1-30.4] | 0.5  [0.4-0.6] | 21.8  [21.2-22.4] | 5.8  [5.4-6.1] | 0.3  [0.3-0.4] | 1.8  [1.6-2.0] |
| Nagaland | 22.4  [21.6-23.2] | 13.8  [13.2-14.5] | 0.2  [0.1-0.3] | 2.6  [2.3-2.9] | 0.2  [0.1-0.3] | 5.6  [5.2-6.1] |
| Manipur | 16.2  [15.4-17] | 0.6  [0.4-0.8] | 5.2  [4.7-5.7] | 4.9  [4.4-5.4] | 0.7  [0.5-0.9] | 5.1  [4.6-5.6] |
| Mizoram | 50.7  [49.5-51.8] | 42.1  [41.0-43.3] | 2.2  [1.8-2.5] | 3.8  [3.3-4.2] | 0.4  [0.2-0.5] | 3.1  [2.7-3.5] |
| Tripura | 37.4  [36.1-38.6] | 19.2  [18.2-20.2] | 0.2  [0.1-0.3] | 0.4  [0.2-0.5] | 0  [0-0.1] | 17.8  [16.8-18.8] |
| Meghalaya | 69.2  [68.3-70.1] | 7.2  [6.7-7.7] | 52.0  [51.0-53.0] | 5.1  [4.7-5.6] | 0  [0-0] | 5.3  [4.9-5.8] |
| Assam | 67.6  [67-68.2] | 0.6  [0.5-0.7] | 0.4  [0.3-0.5] | 1.9  [1.7-2.1] | 0.3  [0.2-0.4] | 64.9  [64.2-65.5] |
| West Bengal | 35.1  [34.3-35.9] | 13.0  [12.4-13.5] | 15.1  [14.5-15.7] | 3.9  [3.6-4.2] | 0.5  [0.4-0.7] | 4.0  [3.7-4.3] |
| Jharkhand | 50.2  [49.6-50.9] | 2.0  [1.8-2.1] | 0.5  [0.4-0.6] | 3.2  [2.9-3.4] | 0.3  [0.2-0.4] | 44.4  [43.7-45] |
| Odisha | 48.2  [47.5-48.8] | 15.8  [15.3-16.2] | 32.6  [32.1-33.2] | 2.5  [2.3-2.6] | 0.8  [0.7-0.9] | 1.6  [1.4-1.7] |
| Chhattisgarh | 71.3  [70.7-71.9] | 54.2  [53.5-54.8] | 6.7  [6.4-7.0] | 2.5  [2.3-2.7] | 0  [0-0.1] | 10.2  [9.8-10.6] |
| Madhya Pradesh | 38.2  [37.7-38.6] | 5.3  [5.1-5.5] | 2.0  [1.9-2.1] | 6.1  [5.9-6.3] | 0.7  [0.6-0.8] | 24.5  [24.1-25] |
| Gujarat | 44.6  [44-45.2] | 6.9  [6.6-7.2] | 25.5  [25.0-26.0] | 8.7  [8.4-9] | 1.5  [1.4-1.7] | 6.9  [6.6-7.2] |
| Dadra & Nagar Haveli And Da.. | 56.6  [54.7-58.4] | 0.0  [0-0.1] | 1.2  [0.7-1.6] | 3.2  [2.5-3.9] | 4.9  [4-5.7] | 49.7  [47.8-51.6] |
| Maharashtra | 22.4  [21.9-22.8] | 2.2  [2-2.4] | 3.3  [3.1-3.5] | 6.5  [6.2-6.8] | 3.1  [2.9-3.3] | 7.6  [7.3-7.9] |
| Andhra Pradesh | 80.1  [79.4-80.9] | 0.8  [0.6-1] | 75.9  [75.1-76.7] | 5.1  [4.7-5.5] | 2.1  [1.9-2.4] | 1.7  [1.4-1.9] |
| Karnataka | 32.1  [31.5-32.7] | 5.4  [5.2-5.7] | 3.4  [3.1-3.6] | 6.3  [6-6.6] | 0.4  [0.3-0.5] | 17.4  [16.9-17.9] |
| Goa | 73.1  [70.6-75.7] | 0.0  [0-0] | 54.8  [51.9-57.6] | 9.3  [7.7-11] | 1.8  [1-2.6] | 10.1  [8.4-11.8] |
| Lakshadweep | 66.9  [63.8-70.1] | 8.3  [6.5-10.1] | 0.3  [0-0.7] | 12.3  [10.1-14.5] | 4.7  [3.3-6.1] | 41.5  [38.3-44.8] |
| Kerala | 58.2  [57.3-59.1] | 45.7  [44.8-46.6] | 2.5  [2.3-2.8] | 4.6  [4.2-4.9] | 3.9  [3.5-4.2] | 2.7  [2.4-2.9] |
| Tamil Nadu | 66.5  [66-67.1] | 0.1  [0.1-0.2] | 56.6  [56.1-57.2] | 15.8  [15.4-16.3] | 1.5  [1.3-1.6] | 0.3  [0.2-0.3] |
| Puducherry | 30.2  [28.7-31.7] | 0.2  [0-0.3] | 15.0  [13.8-16.2] | 16.1  [14.9-17.3] | 2.5  [2.0-3.0] | 0.3  [0.1-0.4] |
| Andaman & Nicobar Islands | 1.8  [1.2-2.3] | 0  [0-0.2] | 0  [0-0.1] | 1.0  [0.6-1.4] | 0.2  [0-0.4] | 0.3  [0.1-0.6] |
| Telangana | 69.2  [68.7-69.8] | 0.1  [0-0.1] | 64.4  [63.8-65] | 4.5  [4.3-4.8] | 0.8  [0.7-0.9] | 0.4  [0.3-0.5] |
| Ladakh | 16.9  [14.9-19] | 0.6  [0.2-1.1] | 4.3  [3.2-5.4] | 11.9  [10.2-13.7] | 0.3  [0-0.7] | 0.2  [0-0.4] |

# Table S5 Percentage of households with at least one member covered by health insurance (and its types) across urban areas in states of India, 2020-21

| **States** | **Any health insurance** | **RSBY** | **State** | **Employer** | **Privately purchased** | **Other** |
| --- | --- | --- | --- | --- | --- | --- |
| Jammu & Kashmir | 21.7  [20.2-23.2] | 3.1  [1.7-4.5] | 14.5  [11.6-17.4] | 67.3  [63.5-71.1] | 7.5  [5.3-9.6] | 11.3  [8.7-13.9] |
| Himachal Pradesh | 41.0  [37.8-44.2] | 2.7  [1-4.3] | 12.3  [9-15.7] | 51.8  [46.7-56.9] | 4.4  [2.3-6.5] | 28.8  [24.2-33.5] |
| Punjab | 27.4  [26.3-28.5] | 12.3  [10.7-13.9] | 23.8  [21.8-25.9] | 37.6  [35.2-40] | 12.9  [11.2-14.5] | 13.8  [12.1-15.5] |
| Chandigarh | 32.2  [28.8-35.5] | 5.9  [3-8.8] | 21.0  [15.9-26] | 37.2  [31.2-43.2] | 29.9  [24.2-35.6] | 7.7  [4.4-11.1] |
| Uttarakhand | 61.6  [59.6-63.6] | 1.8  [1.1-2.5] | 4.4  [3.3-5.4] | 21.8  [19.7-24] | 4.7  [3.6-5.8] | 74.1  [71.9-76.4] |
| Haryana | 28.3  [27.1-29.5] | 9.4  [7.9-10.8] | 6.8  [5.5-8] | 36.5  [34.1-38.8] | 20.3  [18.3-22.2] | 28.4  [26.2-30.6] |
| Nct Of Delhi | 25.1  [24.2-26] | 1.1  [0.7-1.6] | 5.5  [4.5-6.4] | 48.5  [46.5-50.6] | 43.1  [41.1-45.1] | 3.9  [3.1-4.7] |
| Rajasthan | 80.2  [79.3-81.2] | 3.1  [2.7-3.6] | 89.5  [88.7-90.3] | 7.5  [6.8-8.3] | 3.7  [3.2-4.2] | 0.8  [0.5-1.0] |
| Uttar Pradesh | 16.7  [16.1-17.4] | 3.7  [3-4.5] | 3.5  [2.8-4.3] | 21.7  [20-23.3] | 7.4  [6.4-8.5] | 63.8  [61.9-65.8] |
| Bihar | 13.3  [12.3-14.4] | 10.6  [8.0-13.2] | 4.0  [2.3-5.7] | 22.9  [19.3-26.4] | 2.3  [1-3.5] | 60.9  [56.8-65.1] |
| Sikkim | 32.7  [28.9-36.6] | 2.1  [0-4.2] | 14.8  [9.6-20] | 38.6  [31.5-45.7] | 41.0  [33.8-48.2] | 3.0  [0.5-5.5] |
| Arunachal Pradesh | 34.6  [33-36.2] | 1.0  [0.4-1.6] | 65.8  [63.1-68.4] | 23.8  [21.4-26.2] | 2.4  [1.6-3.3] | 8.4  [6.8-9.9] |
| Nagaland | 16.4  [14.9-18] | 62.0  [57.3-66.7] | 1.4  [0.3-2.6] | 15.3  [11.8-18.8] | 0.9  [0-1.9] | 21.6  [17.6-25.5] |
| Manipur | 14.0  [12.5-15.6] | 3.6  [1.4-5.9] | 27.4  [22.1-32.6] | 22.4  [17.4-27.3] | 9.9  [6.4-13.5] | 39.6  [33.8-45.3] |
| Mizoram | 45.5  [43.8-47.3] | 77.6  [75.6-79.7] | 4.7  [3.7-5.8] | 11.5  [9.9-13] | 1.5  [0.9-2.1] | 6.2  [5.0-7.4] |
| Tripura | 27.8  [25.2-30.4] | 43.2  [37.8-48.6] | 0.8  [0.1-1.8] | 1.6  [0.2-3] | 0.5  [-0.2-1.3] | 54.2  [48.8-59.6] |
| Meghalaya | 56.4  [53.5-59.3] | 7.3  [5.4-9.2] | 84.4  [81.8-87] | 5.9  [4.2-7.6] | 0  [0-0] | 2.2  [1.2-3.3] |
| Assam | 56.6  [54.9-58.4] | 1.4  [0.9-2] | 2.3  [1.6-3] | 10.3  [8.8-11.7] | 2.6  [1.8-3.3] | 85.1  [83.5-86.8] |
| West Bengal | 29.1  [27.7-30.4] | 29.3  [26.7-31.9] | 37.3  [34.5-40.1] | 20.2  [17.9-22.6] | 5.0  [3.8-6.3] | 9.9  [8.2-11.6] |
| Jharkhand | 41.5  [40-42.9] | 2.6  [1.8-3.3] | 3.4  [2.6-4.2] | 19.7  [17.9-21.6] | 1.9  [1.3-2.5] | 72.6  [70.6-74.7] |
| Odisha | 29.8  [28.3-31.3] | 25.7  [23.1-28.4] | 42.4  [39.4-45.4] | 22.9  [20.3-25.4] | 9.6  [7.8-11.4] | 4.6  [3.4-5.9] |
| Chhattisgarh | 68.9  [67.5-70.3] | 72.7  [71.1-74.3] | 7.0  [6-7.9] | 7.7  [6.7-8.7] | 0.3  [0.1-0.5] | 17.3  [15.9-18.6] |
| Madhya Pradesh | 41.8  [40.8-42.8] | 11.3  [10.2-12.3] | 4.3  [3.6-5] | 26.3  [24.8-27.7] | 4.7  [4-5.3] | 55.2  [53.6-56.8] |
| Gujarat | 40.6  [39.6-41.6] | 9.9  [8.9-10.8] | 56.8  [55.2-58.3] | 20.4  [19.1-21.7] | 5.9  [5.1-6.6] | 15.7  [14.5-16.9] |
| Dadra & Nagar Haveli And Da.. | 42.9  [40.4-45.4] | 0.2  [-0.1-0.5] | 2.9  [1.6-4.1] | 7.5  [5.6-9.5] | 14.2  [11.6-16.7] | 81.7  [78.9-84.6] |
| Maharashtra | 21.8  [21-22.6] | 7.7  [6.6-8.8] | 12.9  [11.5-14.3] | 35.4  [33.4-37.4] | 25.2  [23.4-27.1] | 20.9  [19.2-22.7] |
| Andhra Pradesh | 69.6  [68-71.2] | 1.6  [1-2.1] | 87.5  [86.1-88.8] | 13.0  [11.6-14.3] | 4.0  [3.1-4.8] | 3.0  [2.3-3.8] |
| Karnataka | 32.0  [31-33.1] | 11.5  [10.2-12.8] | 14.1  [12.7-15.5] | 24.7  [23-26.4] | 1.6  [1.1-2.1] | 51.0  [49-53] |
| Goa | 69.5  [66.1-72.9] | 0  [0-0] | 65.2  [61-69.4] | 16.3  [13-19.5] | 3.8  [2.1-5.5] | 19.5  [16.0-23.0] |
| Lakshadweep | 65.7  [62.1-69.3] | 13.8  [10.6-17] | 0.7  [0-1.4] | 22.2  [18.3-26.1] | 5.3  [3.2-7.4] | 58.5  [53.9-63.1] |
| Kerala | 53.4  [52-54.8] | 72.0  [70.3-73.8] | 4.7  [3.9-5.6] | 9.7  [8.6-10.9] | 9.9  [8.8-11.1] | 6.2  [5.3-7.2] |
| Tamil Nadu | 61.2  [60.3-62.1] | 0.3  [0.2-0.4] | 78.7  [77.7-79.6] | 28.3  [27.3-29.3] | 4.0  [3.6-4.5] | 0.8  [0.6-1] |
| Puducherry | 30.1  [28.4-31.8] | 0.6  [0-1.2] | 52.7  [49.2-56.3] | 54.5  [51.0-58.0] | 9.2  [7.1-11.2] | 1.3  [0.5-2.2] |
| Andaman & Nicobar Islands | 1.2  [0.1-2.2] | 0  [0-0] | 0  [0-0] | 38.6  [9.0-86.4] | 19.4  [-19.3-58.2] | 41.8  [-6.4-90.2] |
| Telangana | 58.4  [57.3-59.6] | 0.1  [0-0.2] | 81.3  [80.1-82.4] | 17.2  [16.1-18.3] | 2.9  [2.4-3.4] | 1.0  [0.7-1.3] |
| Ladakh | 29.5  [23.6-35.5] | 7.9  [1.2-14.6] | 12.0  [4-20.1] | 78.0  [67.8-88.3] | 5.3  [-0.1-10.9] | 1.8  [1.4-5.2] |

# Table S6 Percentage of households with at least one member covered by health insurance (and its types) across rural areas in states of India, 2020-21

| **States** | **Any health insurance** | **RSBY** | **State** | **Employer** | **Privately purchased** | **Other** |
| --- | --- | --- | --- | --- | --- | --- |
| Jammu & Kashmir | 11.5  [10.9-12] | 2.8  [2-3.7] | 15.0  [13.1-16.8] | 68.9  [66.4-71.3] | 4.0  [3-5.1] | 10.9  [9.3-12.5] |
| Himachal Pradesh | 38.4  [37.5-39.4] | 14.1  [12.9-15.2] | 21.9  [20.5-23.2] | 22.4  [21-23.7] | 1.7  [1.3-2.2] | 42.3  [40.7-43.9] |
| Punjab | 23.6  [22.9-24.3] | 16.0  [14.7-17.3] | 29.3  [27.7-31] | 35.2  [33.5-36.9] | 6.3  [5.4-7.1] | 14.9  [13.6-16.2] |
| Chandigarh | 23.8  [5.1-42.4] | 0  [0-0] | 60.0  [11.9-108] | 40.0  [8.0-88] | 0  [0-0] | 0  [0-0] |
| Uttarakhand | 62.8  [61.9-63.8] | 4.1  [3.6-4.6] | 4.4  [3.9-4.9] | 11.0  [10.2-11.7] | 0.4  [0.3-0.6] | 84.4  [83.6-85.3] |
| Haryana | 24.2  [23.4-24.9] | 17.6  [16.2-19] | 5.2  [4.4-6.0] | 23.1  [21.6-24.6] | 5.5  [4.7-6.3] | 49.1  [47.3-50.9] |
| Nct Of Delhi | 26.9  [21.9-31.9] | 1.2  [-1.3-3.7] | 4.8  [0.1-9.7] | 60.7  [49.4-72] | 31.4  [20.7-42.1] | 4.2  [0.4-8.8] |
| Rajasthan | 90.4  [90-90.8] | 3.7  [3.4-3.9] | 95.5  [95.2-95.8] | 2.0  [1.8-2.2] | 0.7  [0.6-0.8] | 0.6  [0.5-0.7] |
| Uttar Pradesh | 15.5  [15.2-15.8] | 7.7  [7.1-8.2] | 1.0  [0.8-1.2] | 6.3  [5.8-6.8] | 1.1  [0.9-1.3] | 84.3  [83.5-85] |
| Bihar | 18.1  [17.7-18.5] | 19.1  [18-20.1] | 1.0  [0.7-1.3] | 8.0  [7.3-8.8] | 0.8  [0.6-1.1] | 71.5  [70.3-72.7] |
| Sikkim | 24.5  [22.9-26.1] | 2.4  [1.3-3.6] | 27.5  [24.1-30.8] | 37.2  [33.6-40.9] | 30.8  [27.3-34.3] | 3.8  [2.3-5.2] |
| Arunachal Pradesh | 28.9  [28.1-29.6] | 1.9  [1.5-2.3] | 74.8  [73.5-76.2] | 18.5  [17.3-19.7] | 1.0  [0.7-1.4] | 5.5  [4.8-6.2] |
| Nagaland | 25.0  [24-26] | 61.8  [59.6-64] | 0.8  [0.4-1.2] | 10.6  [9.2-12.0] | 0.9  [0.5-1.3] | 26.3  [24.4-28.3] |
| Manipur | 17.4  [16.4-18.4] | 4.2  [3.0-5.4] | 34.6  [31.7-37.5] | 34.0  [31.1-36.9] | 2.4  [1.4-3.3] | 27.7  [25-30.4] |
| Mizoram | 57.0  [55.5-58.5] | 88.5  [87.2-89.7] | 3.9  [3.1-4.7] | 3.6  [2.9-4.4] | 0.1  [0-0.3] | 6.0  [5.1-7.0] |
| Tripura | 41.3  [39.9-42.6] | 53.6  [51.4-55.7] | 0.6  [0.3-1.0] | 0.9  [0.5-1.3] | 0  [0-0.2] | 45.9  [43.7-48] |
| Meghalaya | 72.6  [71.7-73.6] | 11.0  [10.2-11.8] | 73.1  [72.0-74.2] | 7.8  [7.1-8.4] | 0  [0-0.1] | 8.9  [8.2-9.5] |
| Assam | 70.0  [69.4-70.7] | 0.8  [0.7-1] | 0.3  [0.2-0.4] | 1.5  [1.3-1.7] | 0.1  [0.1-0.2] | 97.9  [97.6-98.1] |
| West Bengal | 38.1  [37.1-39] | 39.7  [38.2-41.3] | 45.3  [43.7-46.9] | 7.7  [6.9-8.6] | 0.4  [0.2-0.6] | 12.1  [11.1-13.2] |
| Jharkhand | 53.0  [52.3-53.7] | 4.3  [3.9-4.7] | 0.4  [0.3-0.5] | 3.0  [2.7-3.4] | 0.3  [0.2-0.4] | 92.2  [91.6-92.7] |
| Odisha | 51.8  [51.1-52.4] | 33.6  [32.7-34.4] | 70.7  [69.8-71.5] | 3.1  [2.8-3.5] | 0.9  [0.7-1.0] | 3.1  [2.8-3.4] |
| Chhattisgarh | 72.0  [71.3-72.6] | 76.8  [76.1-77.5] | 10.1  [9.6-10.6] | 2.4  [2.1-2.6] | 0  [0-0] | 13.5  [12.9-14] |
| Madhya Pradesh | 36.8  [36.3-37.4] | 15.2  [14.5-15.8] | 5.8  [5.3-6.2] | 11.9  [11.3-12.4] | 0.7  [0.6-0.9] | 68.0  [67.2-68.9] |
| Gujarat | 47.5  [46.9-48.2] | 19.2  [18.4-20] | 57.6  [56.6-58.6] | 18.9  [18.2-19.7] | 1.9  [1.6-2.2] | 15.5  [14.8-16.3] |
| Dadra & Nagar Haveli And Da.. | 72.1  [69.4-74.7] | 0  [-0.1-0.1] | 1.6  [0.7-2.5] | 4.5  [3.0-6.0] | 4.9  [3.4-6.4] | 91.9  [90-93.8] |
| Maharashtra | 22.9  [22.3-23.5] | 11.9  [11-12.8] | 16.4  [15.4-17.4] | 24.2  [23-25.3] | 4.7  [4.1-5.3] | 45.3  [44-46.7] |
| Andhra Pradesh | 84.8  [84-85.6] | 0.8  [0.6-1] | 97.3  [96.9-97.7] | 4.0  [3.5-4.5] | 2.2  [1.9-2.5] | 1.7  [1.4-2] |
| Karnataka | 32.2  [31.5-32.8] | 20.8  [19.7-21.8] | 8.1  [7.4-8.8] | 16.4  [15.5-17.3] | 1.1  [0.9-1.4] | 56.4  [55.2-57.7] |
| Goa | 78.7  [74.9-82.5] | 0  [0-0] | 87.8  [84.4-91.2] | 8.0  [5.2-10.9] | 0.7  [0.1-1.6] | 6.2  [3.7-8.7] |
| Lakshadweep | 71.5  [65.2-77.8] | 7.9  [3.3-12.5] | 0  [0-0] | 5.9  [1.9-9.9] | 12.5  [6.8-18.1] | 73.5  [66.0-81.1] |
| Kerala | 62.5  [61.4-63.7] | 83.4  [82.3-84.5] | 4.1  [3.6-4.7] | 6.4  [5.7-7.2] | 4.2  [3.6-4.8] | 3.3  [2.8-3.9] |
| Tamil Nadu | 71.4  [70.7-72.1] | 0.2  [0.1-0.3] | 90.2  [89.6-90.7] | 20.3  [19.6-21.1] | 0.8  [0.6-1.0] | 0.1  [0-0.2] |
| Puducherry | 30.4  [27-33.8] | 0.9  [-0.3-2.2] | 43.2  [36.5-49.9] | 50.9  [44.1-57.6] | 6.7  [3.3-10.1] | 0.2  [-0.4-0.8] |
| Andaman & Nicobar Islands | 2.1  [1.5-2.8] | 6.2  [-0.6-13.2] | 5.4  [1.0-11.8] | 60.2  [46.2-74.2] | 12.8  [3.2-22.4] | 15.1  [4.9-25.4] |
| Telangana | 75.0  [74.4-75.6] | 0.1  [0-0.2] | 97.9  [97.7-98.1] | 2.1  [1.8-2.3] | 0.5  [0.4-0.6] | 0.4  [0.3-0.5] |
| Ladakh | 14.5  [12.4-16.7] | 2.3  [0-4.6] | 30.6  [23.4-37.9] | 67.6  [60.2-74.9] | 1.1[-0.5-2.7] | 1.1  [0.5-2.8] |

**Table S7 Percentage of females aged 15 to 49 years covered by health insurance across demographic and socioeconomic categories in India, 2020-21**

|  | **% (95% CI)** | | | | | |
| --- | --- | --- | --- | --- | --- | --- |
|  | **Any health insurance** | **RSBY** | **State** | **Employer** | **Privately purchased** | **Other** |
|  |  |  | | | | |
| India | 29.7  [29.6-29.8] | 16.4  [16.2-16.5] | 48.8  [48.6-49] | 9.8  [9.7-9.9] | 2.3  [2.3-2.4] | 25.5  [25.3-25.7] |
|  |  |  |  |  |  |  |
| Marital status |  |  |  |  |  |  |
| Currently unmarried | 28.6  [28.4-28.8] | 16.7  [16.4-17] | 49.5  [49.1-49.9] | 9.1  [8.8-9.3] | 1.9  [1.8-2.0] | 25.3  [25.0-25.6] |
| Currently married | 30.2  [30-30.3] | 16.3  [16.1-16.5] | 48.5  [48.3-48.7] | 10.1  [9.9-10.2] | 2.5  [2.4-2.6] | 25.5  [25.3-25.8] |
|  |  |  |  |  |  |  |
| Years of education |  |  |  |  |  |  |
| Illiterate | 32.5  [32.3-32.7] | 14.0  [13.7-14.2] | 55.0  [54.6-55.4] | 5.4  [5.2-5.5] | 0.3  [0.3-0.4] | 27.7  [27.4-28.1] |
| 1 to 4 years | 33.3  [32.8-33.8] | 18.3  [17.6-18.9] | 49.8  [49.0-50.7] | 7.4  [6.9-7.8] | 0.6  [0.5-0.7] | 28.3  [27.5-29.0] |
| 5 to 9 years | 29.3  [29.1-29.5] | 17.4  [17.1-17.7] | 48.2  [47.8-48.5] | 8.0  [7.8-8.2] | 0.9  [0.8-1.0] | 28.4  [28.1-28.7] |
| 10 to 12 years | 27.8  [27.6-28] | 18.7  [18.3-19] | 46.6  [46.2-47.0] | 11.3  [11.0-11.6] | 2.7  [2.5-2.8] | 23.3  [23.0-23.7] |
| 12 years or more | 28.3  [28-28.6] | 14.0  [13.6-14.4] | 42.8  [42.2-43.3] | 19.4  [18.9-19.8] | 8.8  [8.5-9.1] | 18.0  [17.6-18.4] |
|  |  |  |  |  |  |  |
| Age |  |  |  |  |  |  |
| 15 to 30 | 25.3  [25.1-25.4] | 16.1  [15.8-16.3] | 49.8  [49.5-50.1] | 8.9  [8.7-9.1] | 1.9  [1.8-1.9] | 25.6  [25.4-25.9] |
| 30 to 44 | 33.3  [33.1-33.5] | 16.4  [16.2-16.6] | 47.6  [47.3-47.9] | 10.5  [10.3-10.7] | 2.7  [2.6-2.8] | 25.9  [25.6-26.1] |
| 45 to 59 | 36.7  [36.3-37] | 17.3  [16.9-17.8] | 49.3  [48.7-49.8] | 10.4  [10.0-10.7] | 2.5  [2.4-2.7] | 23.9  [23.4-24.3] |
|  |  |  |  |  |  |  |
| Religion |  |  |  |  |  |  |
| Hindu | 30.9  [30.8-31.1] | 16.0  [15.8-16.1] | 49.9  [49.6-50.1] | 10.1  [9.9-10.2] | 2.3  [2.3-2.4] | 24.7  [24.5-24.9] |
| Muslim | 22.0  [21.8-22.3] | 19.5  [18.9-20] | 40.1  [39.5-40.8] | 5.8  [5.5-6.1] | 0.9  [0.8-1.1] | 35.2  [34.5-35.8] |
| Others | 30.4  [30.1-30.7] | 17.5  [17.0-18.0] | 47.6  [47.0-48.2] | 12.8  [12.4-13.2] | 5.2  [5.0-5.5] | 19.7  [19.2-20.2] |
|  |  |  |  |  |  |  |
| Caste |  |  |  |  |  |  |
| Scheduled caste | 29.3  [29.2-29.4] | 15.5  [15.4-15.7] | 50.4  [50.2-50.6] | 10.0  [9.9-10.2] | 2.5  [2.4-2.6] | 24.5  [24.3-24.7] |
| Scheduled tribe | 39.3  [39-39.6] | 24.7  [24.3-25.1] | 44.5  [44.0-45.0] | 6.8  [6.5-7.0] | 0.2  [0.2-0.3] | 26.1  [25.7-26.5] |
| No caste /tribe | 26.8  [26.4-27.3] | 17.6  [16.9-18.4] | 24.0  [23.2-24.9] | 10.3  [9.7-10.9] | 2.9  [2.6-3.2] | 45.9  [45.0-46.9] |
| Don’t know | 21.6  [20.3-22.8] | 26.1  [23.2-28.9] | 47.8  [44.6-51.0] | 9.6  [7.7-11.5] | 1.1  [0.4-1.8] | 17.2  [14.8-19.6] |
|  |  |  |  |  |  |  |
| Wealth quintile |  |  |  |  |  |  |
| Lowest | 26.6  [26.4-26.8] | 23.0  [22.6-23.4] | 31.3  [30.9-31.7] | 4.6  [4.4-4.8] | 0.1  [0.0-0.1] | 43.1  [42.6-43.6] |
| Second | 30.6  [30.4-30.8] | 17.4  [17.1-17.8] | 46.9  [46.5-47.3] | 5.6  [5.3-5.8] | 0.2  [0.2-0.2] | 32.3  [31.9-32.8] |
| Third | 32.0  [31.8-32.3] | 15.2  [14.9-15.5] | 57.4  [57.0-57.9] | 7.1  [6.9-7.3] | 0.5  [0.4-0.5] | 22.9  [22.5-23.3] |
| Fourth | 31.4  [31.1-31.6] | 15.9  [15.6-16.3] | 57.7  [57.3-58.2] | 9.5  [9.2-9.7] | 1.1  [1-1.2] | 19.0  [18.6-19.3] |
| Highest | 28.0  [27.8-28.2] | 12.4  [12.1-12.7] | 45.8  [45.3-46.3] | 19.9  [19.5-20.3] | 8.7  [8.4-9.0] | 16.1  [15.7-16.4] |

**Table S8 Percentage of males aged 15 to 54 years covered by health insurance across demographic and socioeconomic categories in India, 2020-21**

|  | **% (95% CI)** | | | | | |
| --- | --- | --- | --- | --- | --- | --- |
|  | **Any health insurance** | **RSBY** | **State** | **Employer** | **Privately purchased** | **Other** |
|  |  |  | | | | |
| **India** | 33.8  [33.6-34.1] | 14.2  [13.8-14.5] | 48.0  [47.5-48.5] | 13.3  [12.9-13.6] | 3.7  [3.5-3.9] | 24.5  [24.0-24.9] |
|  |  |  |  |  |  |  |
| **Marital status** |  |  |  |  |  |  |
| Currently unmarried | 29.7  [29.3-30.2] | 14.5  [13.9-15.1] | 49.2  [48.3-50.1] | 13.3  [12.7-14.0] | 2.7  [2.4-3.0] | 23.4  [22.6-24.2] |
| Currently married | 36.3  [36.0-36.7] | 14.0  [13.6-14.4] | 47.4  [46.8-48.0] | 13.3  [12.8-13.7] | 4.1  [3.9-4.4] | 25.0  [24.5-25.5] |
|  |  |  |  |  |  |  |
| **Years of education** |  |  |  |  |  |  |
| Illiterate | 36.1  [35.3-37.0] | 12.8  [11.8-13.8] | 58.8  [57.4-60.2] | 6.0  [5.4-6.7] | 0.6  [0.4-0.8] | 24.2  [23.0-25.4] |
| 1 to 4 years | 32.6  [31.4-33.9] | 18.3  [16.7-19.9] | 45.0  [42.9-47.1] | 8.0  [6.9-9.1] | 0.7  [0.3-1.1] | 31.4  [29.4-33.3] |
| 5 to 9 years | 32.3  [31.8-32.7] | 16.7  [16.0-17.3] | 048.6  [47.7-49.4] | 8.5  [8.0-9.0] | 1.2  [1.0-1.4] | 28.1  [27.3-28.9] |
| 10 to 12 years | 33.1  [32.6-33.6] | 14.2  [13.5-14.9] | 47.7  [46.7-48.6] | 14.1  [13.4-14.7] | 3.2  [2.9-3.6] | 23.9  [23.1-24.7] |
| 12 years or more | 36.6  [35.9-37.3] | 10.0  [9.3-10.7] | 41.7  [40.6-42.9] | 25.4  [24.4-26.5] | 10.7  [10.0-11.5] | 17.9  [16.9-18.8] |
|  |  |  |  |  |  |  |
| **Age** |  |  |  |  |  |  |
| 15 to 30 | 29.5  [29.1-30.0] | 13.7  [13.1-14.3] | 50.2  [49.4-51.0] | 13.1  [12.5-13.6] | 2.4  [2.2-2.7] | 23.7  [23.0-24.4] |
| 30 to 44 | 35.8  [35.3-36.3] | 13.7  [13.1-14.3] | 47.0  [46.2-47.8] | 13.2  [12.7-13.8] | 4.7  [4.3-5.0] | 25.1  [24.4-25.8] |
| 45 to 54 | 39.9  [39.2-40.6] | 15.7  [15.0-16.5] | 45.9  [44.8-47.0] | 13.8  [13.0-14.5] | 4.1  [3.6-4.5] | 24.7  [23.8-25.6] |
|  |  |  |  |  |  |  |
| **Religion** |  |  |  |  |  |  |
| Hindu | 35.2  [34.8-35.5] | 13.2  [12.8-13.6] | 49.2  [48.6-49.7] | 13.6  [13.2-14.0] | 3.7  [3.5-3.9] | 24.1  [23.7-24.6] |
| Muslim | 25.4  [24.6-26.2] | 19.0  [17.6-20.3] | 41.7  [40.0-43.4] | 8.9  [7.9-9.9] | 1.8  [1.4-2.3] | 30.8  [29.2-32.4] |
| Others | 38.5  [37.7-39.4] | 18.3  [17.1-19.5] | 43.9  [42.4-45.5] | 17.3  [16.1-18.5] | 6.3  [5.6-7.1] | 16.7  [15.6-17.9] |
|  |  |  |  |  |  |  |
| **Caste** |  |  |  |  |  |  |
| Scheduled caste | 34.4  [34.1-34.7] | 13.6  [13.2-14.0] | 49.9  [49.4-50.5] | 12.8  [12.4-13.2] | 3.8  [3.6-4.0] | 23.4  [22.9-23.8] |
| Scheduled tribe | 38.9  [38.2-39.7] | 21.7  [20.7-22.8] | 39.6  [38.3-40.8] | 13.7  [12.8-14.6] | 1.3  [1.0-1.6] | 27.9  [26.7-29.0] |
| No caste /tribe | 25.6  [24.3-26.8] | 13.4  [11.6-15.2] | 26.4  [24.1-28.7] | 20.1  [18.0-22.2] | 4.2  [3.1-5.2] | 38.4  [35.9-40.9] |
| Don’t know | 19.3  [16.1-22.4] | 20.1  [12.7-27.4] | 59.3  [50.3-68.4] | 12.2  [6.1-18.2] | 2.5  [0.3-5.5] | 13.9  [7.5-20.3] |
|  |  |  |  |  |  |  |
| **Wealth quintile** |  |  |  |  |  |  |
| Lowest | 27.7  [27.0-28.3] | 19.6  [18.7-20.5] | 31.1  [30.0-32.2] | 7.2  [6.6-7.8] | 0.5  [0.3-0.6] | 44.2  [43.1-45.4] |
| Second | 32.1  [31.5-32.7] | 17.0  [16.2-17.9] | 46.9  [45.8-48.0] | 6.6  [6.1-7.2] | 0.6  [0.4-0.8] | 32.4  [31.4-33.5] |
| Third | 36.0  [35.3-36.6] | 13.5  [12.7-14.3] | 54.7  [53.6-55.8] | 9.3  [8.7-10.0] | 1.3  [1.0-1.5] | 24.1  [23.1-25.0] |
| Fourth | 38.4  [37.7-39.1] | 14.1  [13.3-14.9] | 56.8  [55.7-57.9] | 12.7  [12.0-13.5] | 2.0  [1.7-2.3] | 17.7  [16.9-18.6] |
| Highest | 33.6  [33.0-34.3] | 9.5  [8.8-10.2] | 43.2  [42.1-44.4] | 26.1  [25.1-27.1] | 11.8  [11.1-12.6] | 14.4  [13.6-15.2] |

**Table S9 Relative risk ratio for coverage under type of health insurance among households that have some form of health insurance, India, 2020-21**

|  | **Rashtriya Swashtya Bima Yojana** | | | **State health insurance schemes** | | **Privately purchased scheme** | | **Other health insurance schemes** | |
| --- | --- | --- | --- | --- | --- | --- | --- | --- | --- |
|  | **relative risk ratio** | **95% CI** | | **relative risk ratio** | **95% CI** | **relative risk ratio** | **95% CI** | **relative risk ratio** | **95% CI** |
| **Wealth** |  |  | |  |  |  |  |  |  |
| Lowest | Ref | Ref | | Ref | Ref | Ref | Ref | Ref | Ref |
| Low | 0.5*** | [0.5-0.5] | | 0.8*** | [0.7-0.8] | 1.1 | [0.8-1.4] | 0.5*** | [0.5-0.5] |
| Middle | 0.4*** | [0.3-0.4] | | 0.8*** | [0.7-0.8] | 1.7*** | [1.3-2.2] | 0.3*** | [0.3-0.3] |
| High | 0.4*** | [0.4-0.4] | | 1 | [0.9-1] | 2.5*** | [2-3.2] | 0.2*** | [0.2-0.2] |
| Highest | 0.3*** | [0.3-0.4] | | 1.8*** | [1.7-1.9] | 8.5*** | [6.7-10.7] | 0.2*** | [0.2-0.2] |
|  |  |  | |  |  |  |  |  |  |
| **Education** | |  | |  |  |  |  |  |  |
| Illiterate | Ref | Ref | | Ref | Ref | Ref | Ref | Ref | Ref |
| 1 to 4 years | 1.8*** | [1.8-1.9] | | 1.4*** | [1.3-1.5] | 1.6*** | [1.4-1.9] | 1.4*** | [1.3-1.4] |
| 5 to 9 years | 1.8*** | [1.8-1.9] | | 1.6*** | [1.5-1.6] | 1.8*** | [1.6-2] | 1.6*** | [1.6-1.7] |
| 10 to 12 years | 1.8*** | [1.7-1.9] | | 2.3*** | [2.2-2.4] | 3.1*** | [2.8-3.5] | 2*** | [1.9-2.1] |
| 12 years or more | 1.4*** | [1.4-1.5] | | 3.6*** | [3.4-3.8] | 6.3*** | [5.6-7.1] | 2*** | [1.9-2.1] |
|  |  |  | |  |  |  |  |  |  |
| **Social Category** |  |  | |  |  |  |  |  |  |
| Scheduled caste | Ref | Ref | | Ref | Ref | Ref | Ref | Ref | Ref |
| Scheduled tribe | 1.7*** | [1.6-1.8] | | 1.1*** | [1-1.1] | 0.8** | [0.7-0.9] | 0.8*** | [0.8-0.8] |
| Other backward castes | 0.8*** | [0.8-0.9] | | 0.7*** | [0.7-0.7] | 1.1** | [1-1.2] | 0.8*** | [0.8-0.8] |
| General | 1.1*** | [1-1.1] | | 1.3*** | [1.3-1.4] | 3.1*** | [2.9-3.5] | 1.5*** | [1.5-1.6] |
| Don’t know | 1.1 | [0.9-1.3] | | 1.2 | [1-1.5] | 2.4*** | [1.7-3.5] | 1 | [0.9-1.2] |
|  |  |  | |  |  |  |  |  |  |
| **Place of residence** | |  | |  |  |  |  |  |  |
| Urban | Ref | Ref | | Ref | Ref | Ref | Ref | Ref | Ref |
| Rural | 0.8*** | [0.8-0.8] | | 0.6 | [0.6-0.7] | 0.5*** | [0.4-0.5] | 0.9*** | [0.8-0.9] |
|  |  |  | |  |  |  |  |  |  |
| **Age (household head)** | | | |  |  |  |  |  |  |
| 13 to 29 | Ref | Ref | | Ref | Ref | Ref | Ref | Ref | Ref |
| 30 to 44 | 1.1*** | [1.1-1.2] | | 1.2*** | [1.1-1.2] | 1.1 | [0.9-1.2] | 1.1*** | [1-1.1] |
| 45 to 59 | 1.4*** | [1.3-1.5] | | 1.4*** | [1.3-1.5] | 1.2** | [1-1.4] | 1.2*** | [1.2-1.3] |
| 60 to 74 | 1.5*** | [1.4-1.6] | | 1.5*** | [1.4-1.6] | 1.2** | [1-1.4] | 1.2*** | [1.2-1.3] |
| 75 and above | 1.7*** | [1.6-1.8] | | 1.7*** | [1.5-1.8] | 1.6*** | [1.4-2] | 1.2*** | [1.1-1.3] |
|  |  |  | |  |  |  |  |  |  |
| **Religion** |  |  | |  |  |  |  |  |  |
| Hindu | Ref | Ref | | Ref | Ref | Ref | Ref | Ref | Ref |
| Muslim | 1.2*** | [1.1-1.3] | | 1.2*** | [1.1-1.2] | 0.7*** | [0.6-0.8] | 1.4*** | [1.3-1.4] |
| Others | 1.1*** | [1.1-1.2] | | 1.1*** | [1-1.1] | 1.5*** | [1.3-1.6] | 0.5*** | [0.5-0.5] |
|  |  |  | |  |  |  |  |  |  |
| **Household size** | |  | |  |  |  |  |  |  |
| Less than 4 | Ref | Ref | | Ref | Ref | Ref | Ref | Ref | Ref |
| More than 4 | 1*** | [1-1.1] | | 1 | [0.9-1] | 1.1*** | [1-1.1] | 1.3*** | [1.3-1.4] |
|  |  |  | |  |  |  |  |  |  |
| **Gender (household head)** | | | |  |  |  |  |  |  |
| Male | Ref | | Ref | Ref | Ref | Ref | Ref | Ref | Ref |
| Female | 0.9*** | | [0.8-0.9] | 1 | [0.9-1] | 0.9 | [0.8-1] | 1.1*** | [1.1-1.1] |
|  |  | |  |  |  |  |  |  |  |
| **Marital Status** | | |  |  |  |  |  |  |  |
| Currently unmarried | Ref | | Ref | Ref | Ref | Ref | Ref | Ref | Ref |
| Currently married | 0.8*** | | [0.7-0.8] | 0.9 | [0.9-1] | 0.7*** | [0.7-0.8] | 1*** | [1-1.1] |


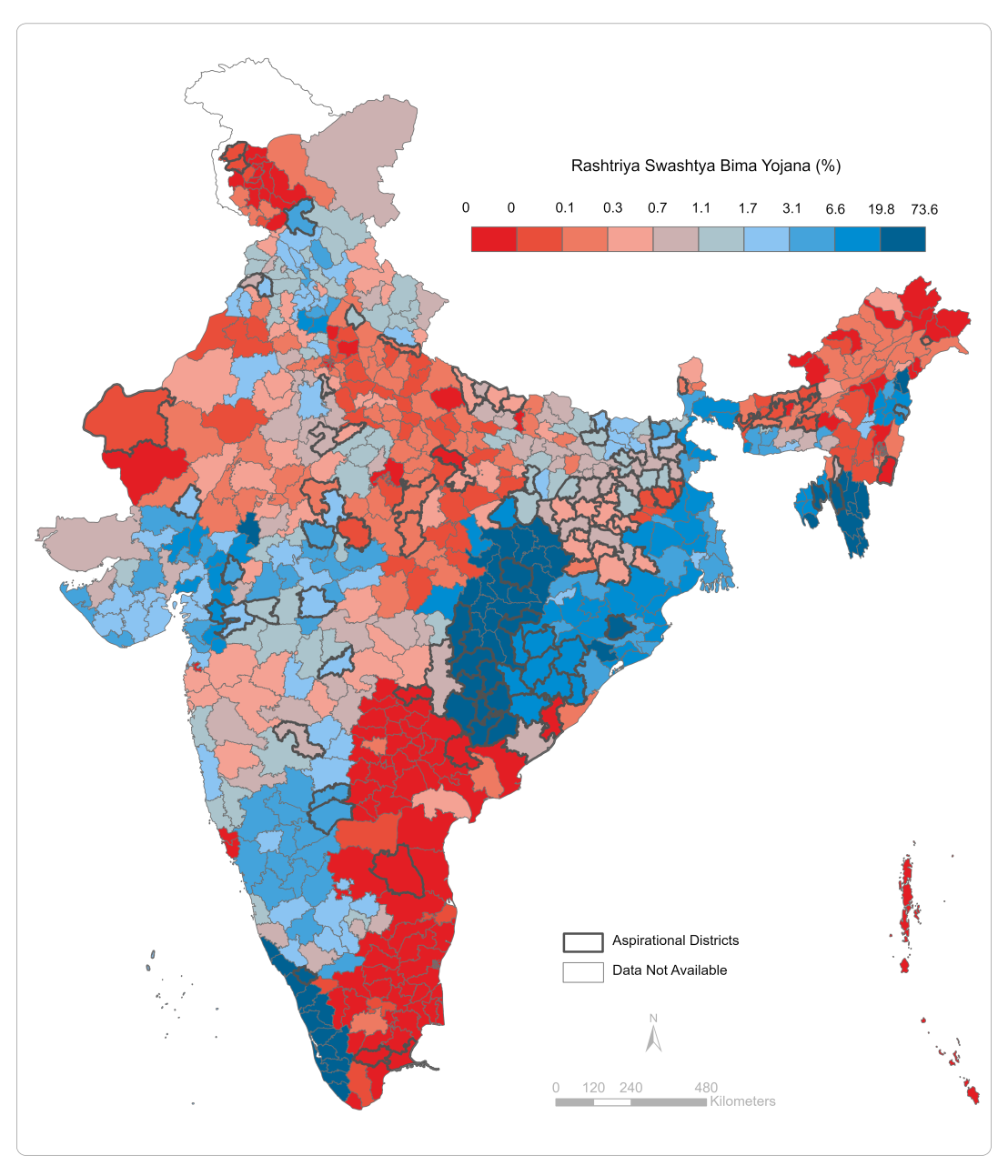

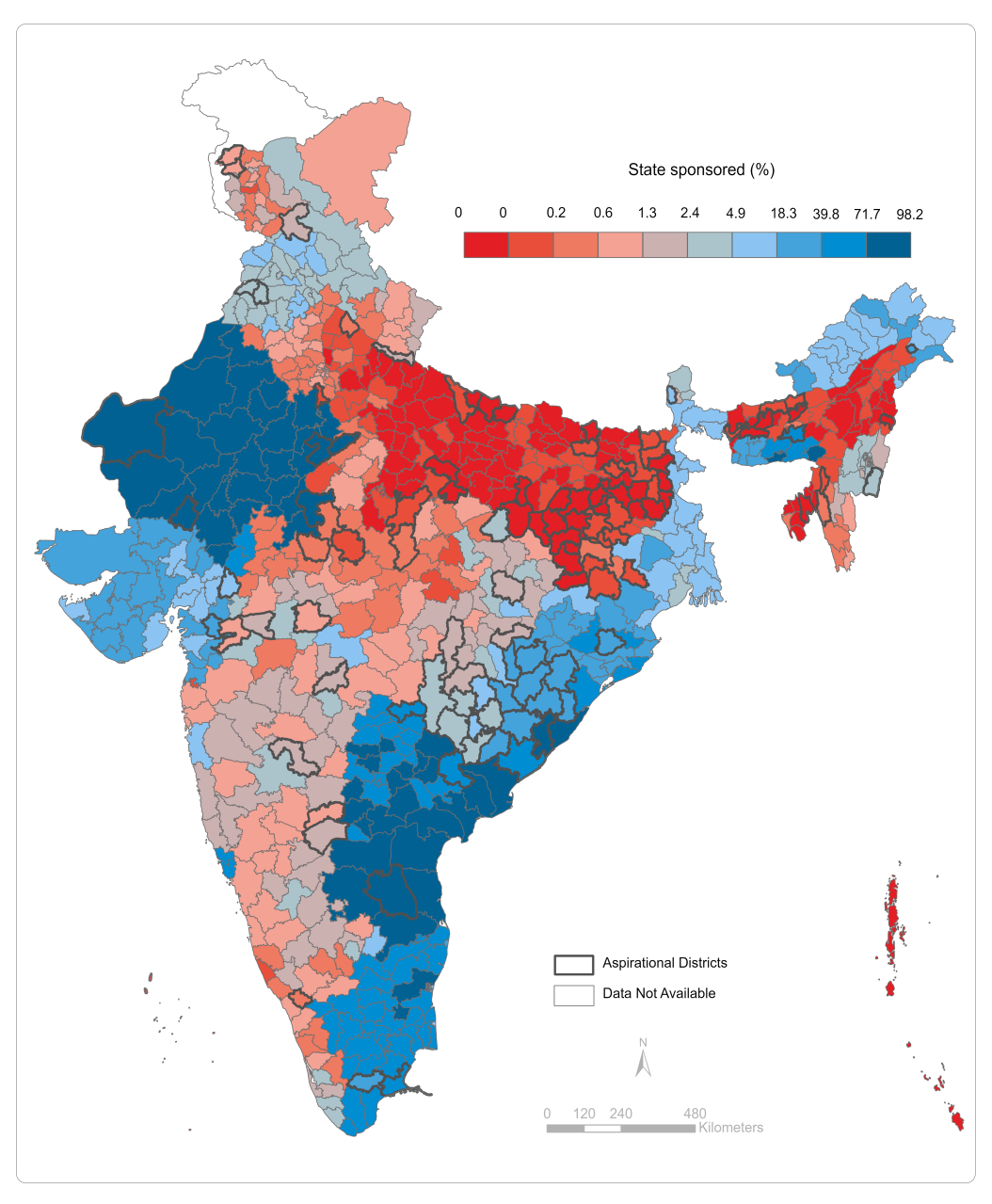


Figure S1 Percentage of households covered by RSBY in districts of India, 2020-21

\

Figure S2 Percentage of households covered by state health insurance schemes in districts of India, 2020-21


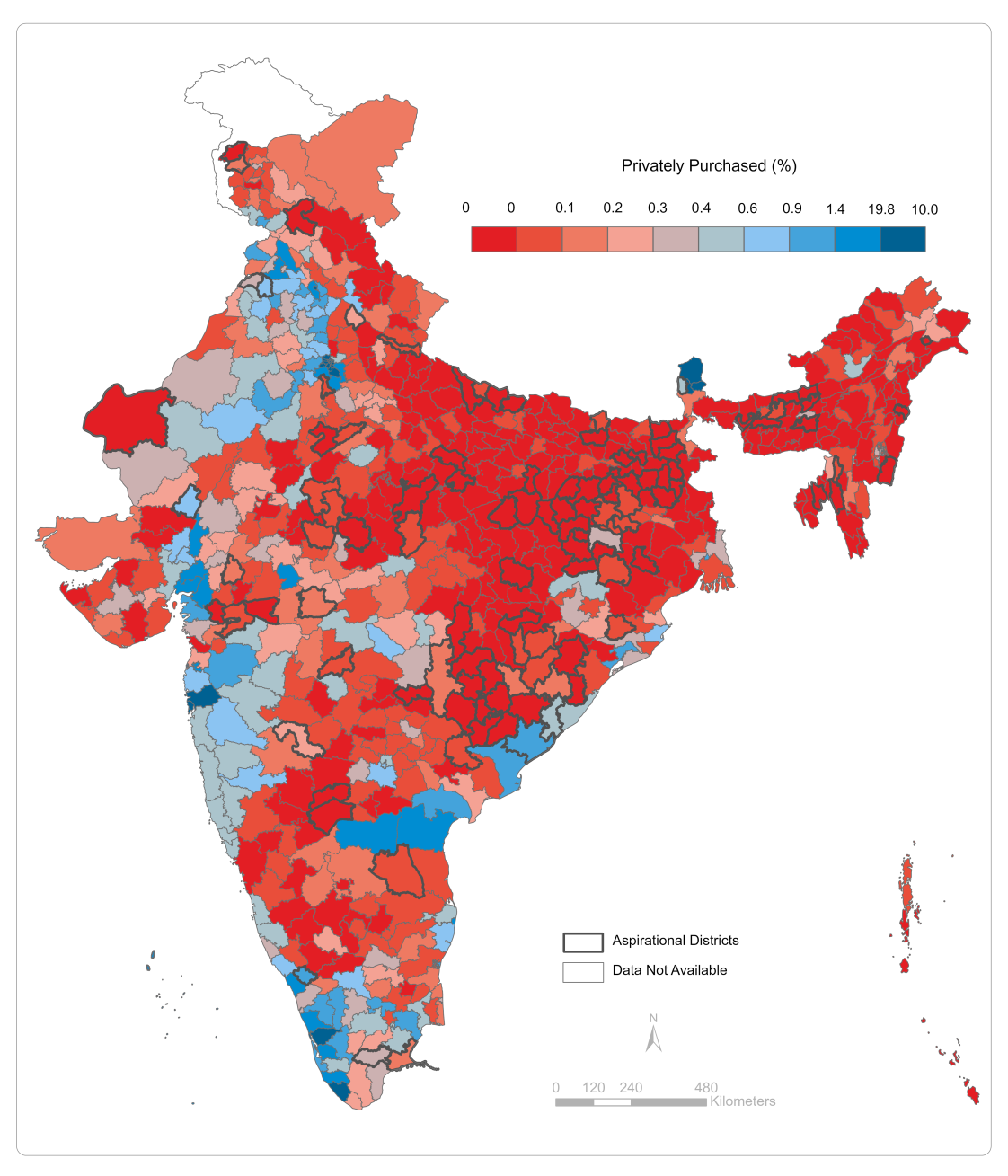

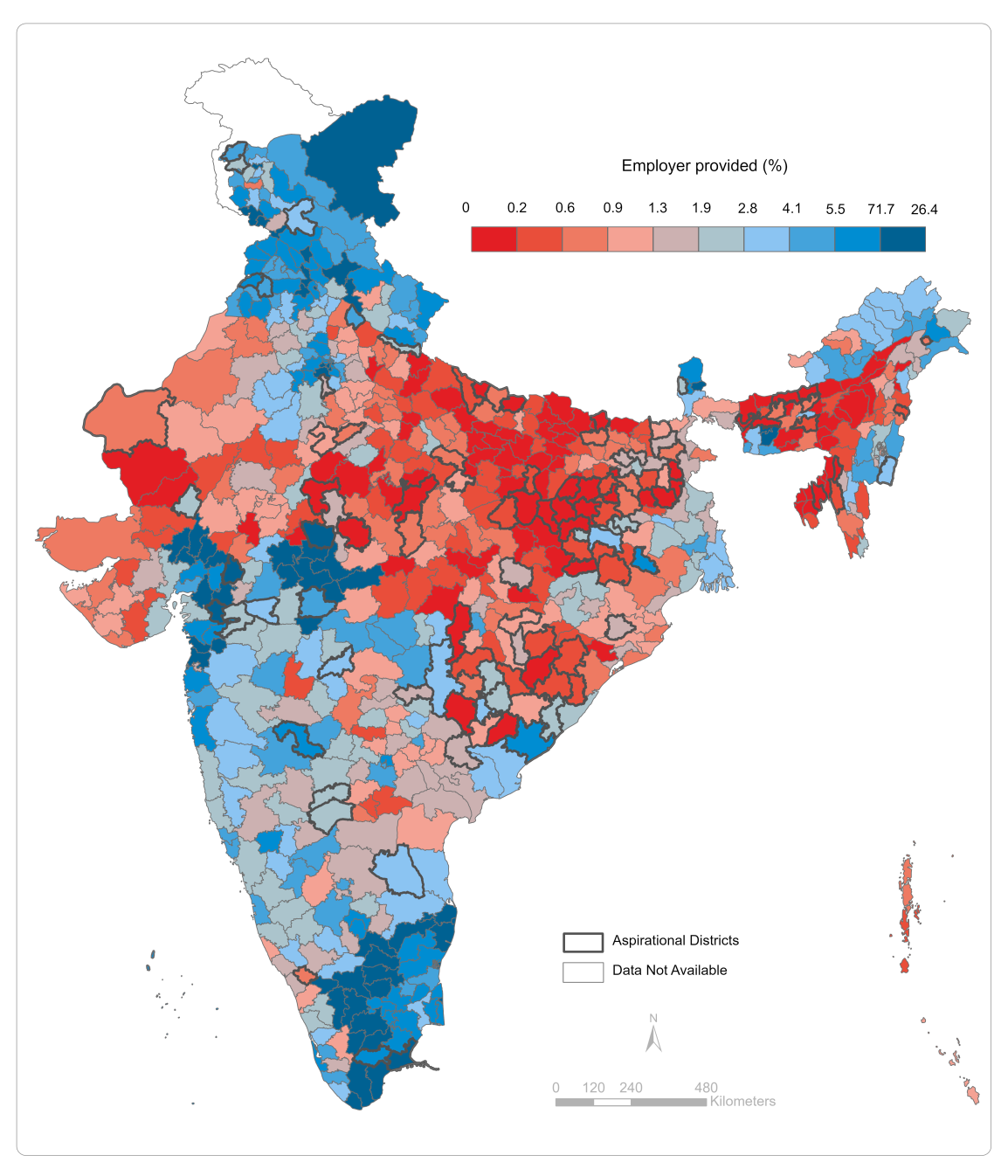


Figure S4 Percentage of households covered by employer provided health insurance in districts of India, 2020-21

Figure S3 Percentage of households covered by privately purchased health insurance in districts of India, 2020-21

Figure S4 Percentage of households covered by privately purchased health insurance in districts of India, 2019-20

Figure S5 Percentage of households covered by employer-provided health insurance in districts of India, 2019-20

**
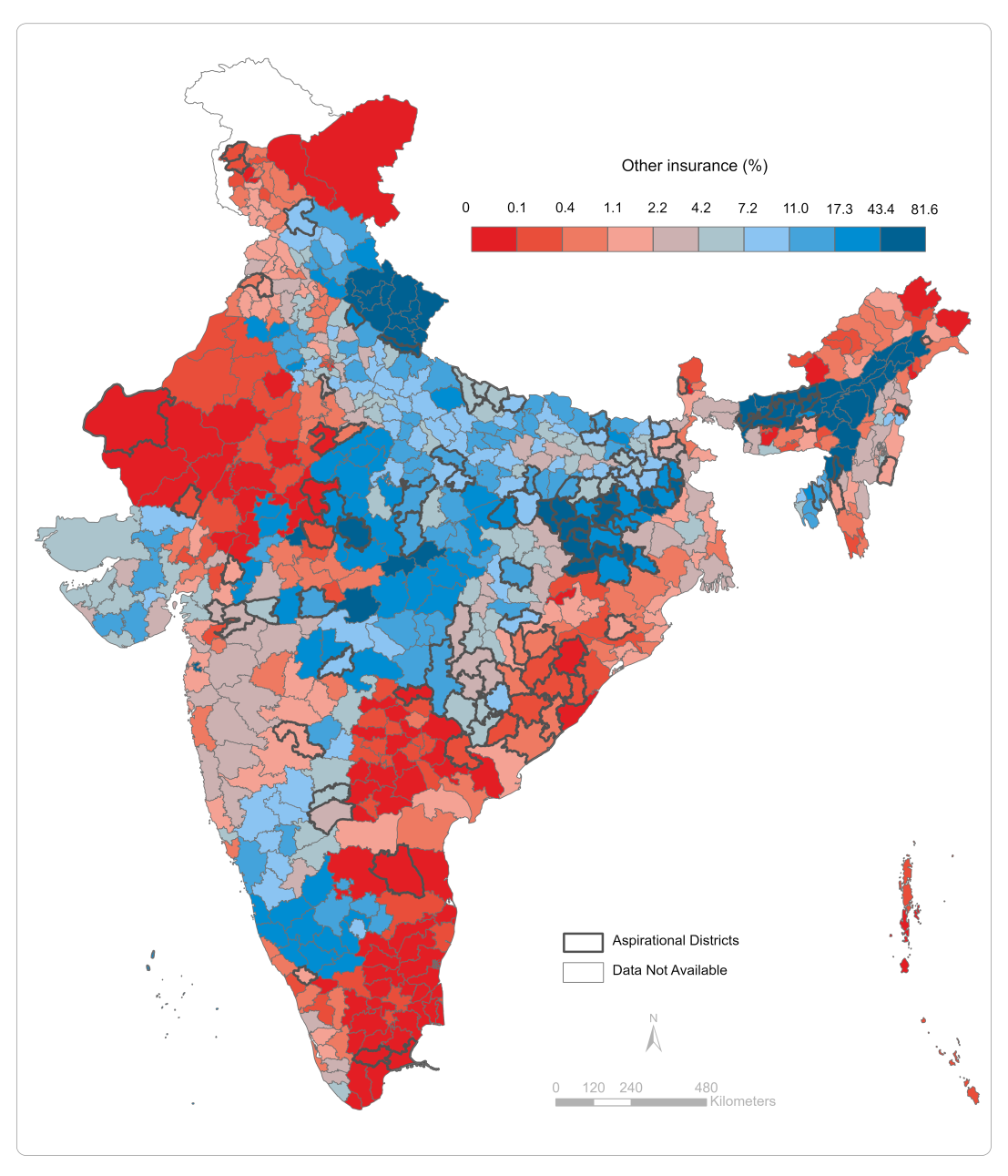
**

Figure S5 Percentage of households covered by “other” health insurance in districts of India, 2020-21
